# Distinct developmental trajectories of health-related quality of life for boys and girls throughout childhood and adolescence; a national level longitudinal study

**DOI:** 10.1186/s12955-023-02171-5

**Published:** 2023-08-01

**Authors:** Rachel O’Loughlin, Harriet Hiscock, Nancy Devlin, Kim Dalziel

**Affiliations:** 1 Health Economics Unit, Melbourne School of Population and Global Health, University of Melbourne, Melbourne, VIC 3010 Australia; 2 Department of Paediatrics, Melbourne Medical School, University of Melbourne, Melbourne, VIC 3010 Australia; 3 Health Services Research Unit, The Royal Children’s Hospital, Parkville, VIC 3052 Australia; 4 Health Services and Economics, Murdoch Children’s Research Institute, Parkville, VIC 3052 Australia

**Keywords:** Health-related quality of life, Child, Adolescent, Child development, Outcome assessment, Health care

## Abstract

**Background:**

To identify and describe distinct developmental trajectories of health-related quality of life (HRQoL) in a national level Australian population sample, overall and separately for boys and girls.

**Methods:**

Data were from the *Longitudinal Study of Australian Children* (LSAC). Participants were children aged 4–5 years recruited in 2004 and followed through to age 16–17 years in 2016, and their caregivers. Group-based trajectory modelling was used to identify groups of children that follow qualitatively distinct developmental trajectories of HRQoL.

**Results:**

Three distinct trajectories were identified for the total sample: (1) high-stable (52.2% of children); (2) middle-stable (38.0%); and (3) low-declining (9.8%). These trajectories differed for boys, who saw increasing HRQoL in the highest trajectory group; a middle-stable trajectory; and declining and rebounding HRQoL in the lowest trajectory group. In contrast, girls saw no increasing or rebounding trajectories; approximately half of girls had high-stable HRQoL and the remaining half had either steadily or rapidly declining HRQoL from age 4–5 to 16–17 years.

**Conclusions:**

Our results highlight the importance of considering the distinct trajectories for girls and boys and not relying on population mean levels of HRQoL for decision-making. The presence of developmentally distinct trajectories of HRQoL, and differences in the trajectories faced by boys and girls, should be considered when assessing the effectiveness of treatments and interventions impacting upon HRQoL throughout childhood and adolescence. Failure to account for these pre-existing trajectories may over- or under-estimate treatment effects.

**Supplementary Information:**

The online version contains supplementary material available at 10.1186/s12955-023-02171-5.

## Background

Health-related quality of life (HRQoL) is a multi-dimensional construct that captures the impact of the child’s health status on different aspects of their physical, social and psychological functioning, either through self or proxy report [1]. Understanding a child’s HRQoL is informative for clinical treatment planning, including periodicity of review and exploring wider factors affecting the child’s wellbeing including need for education support or family support. Indeed, some argue it is the “*universal outcome towards which all our efforts regarding children ultimately should be directed” *[1]. Aside from its clinical importance, HRQoL information is also fundamental to health economic analyses, as it allows meaningful comparisons of the outcomes from competing health interventions. Population-level longitudinal studies examining developmentally distinct trajectories of children’s HRQoL remain uncommon, such that it is unclear how HRQoL changes across childhood and adolescence at a population level, and whether there are important subgroups with poorer trajectories that may benefit from specific attention in clinical and health policy decision-making. Early identification and intervention are crucial steps in ameliorating the effects of disadvantage on children’s wellbeing, benefitting not only the child and their own future children, but resulting in economic benefits for wider society [2]. However, in order to understand the true effects of intervention efforts in this space, we first need to understand the natural trajectories of HRQoL throughout childhood and adolescence. Failure to account for these underlying trajectories may lead to over- or under-estimation of intervention effects.

To our knowledge there have only been two previous population-based studies that have aimed to identify distinct developmental trajectories of children’s HRQoL [3, 4], both using the PedsQL HRQoL instrument. In a sample of Australian children recruited at age 4 years (*n* = 2,785) and followed through to 13 years, Vella et al. [3] modelled four distinct trajectories in which the majority of children (85%) had stable, high HRQoL; 8% were considered high risk with rapidly falling HRQoL; 5% saw an initial drop in HRQoL with a subsequent ‘rebound’ period; and 2% had initially low HRQoL that improved gradually through childhood. In a different sample of Australian children aged 4–13 years (*n* = 1,482), Le et al. [4] identified three trajectories of HRQoL: stable-high (51% of children); reduced initial HRQoL with slow decline (40%); and low initial HRQoL with rapid decline (9%).

These two existing studies have several limitations. Both followed children’s trajectories only to age 12–13 years, leaving unknown any changes in trajectory later in adolescence. Additionally, these studies did not examine trajectories separately by sex, though cross-sectional studies suggest HRQoL may generally be lower for girls than boys, particularly later in adolescence [5]. Arising from the limitations of existing evidence, the aim of this study was to identify and describe distinct developmental trajectories of HRQoL in an Australian population sample aged 4–17 years, overall and separately for boys and girls.

## Methods

### Study design & participants

Data were from the *Longitudinal Study of Australian Children*(LSAC), a national level longitudinal study with data collected from participants every two years between 2004–2016 [6]. Children in the current study were aged 4–5 years at recruitment and followed at seven time points to age 16–17 years. Data were additionally recorded for the child’s primary caregiver (“Parent 1”) and their partner (“Parent 2”, if applicable) in order to describe the sample at each Wave. The LSAC study was approved by the Australian Institute of Family Studies Ethics Committee, and families provided written consent to participate. The current study was approved by the data custodians and no further ethics approval was required.

### Measures

#### Primary outcome measure

*Children’s health-related quality of life (HRQoL)*was measured using the Pediatric Quality of Life Inventory (PedsQL), a 23-item generic measure of HRQoL comprising four domains: physical; emotional; social; and school functioning [7], see Supplementary Table 1. The PedsQL is considered to be feasible, valid and reliable in general population research [7]. Parent 1 rated their child’s functioning over the last month on a scale from 1 ‘never a problem’ to 5 ‘almost always a problem’. Items were reverse scored and linearly transformed to a 0–100 scale such that higher scores represent better HRQoL. A change of 4.5 points on the PedsQL total score is considered a clinically important difference using the parent proxy-report form [7].

#### Child, parent and family characteristics

A range of child, parent and family characteristics were collected and are presented to describe the sample at each time point. *Child age (in years)* was recorded at each wave. *Child sex* was recorded at Wave 1, and this value was continued to other waves. *Language other than English (LOTE)* was indicated if the main language spoken by the child at home was any language other than English.

*Children’s mental health symptoms *were measured using the Strengths and Difficulties Questionnaire (SDQ), a widely used and validated screening instrument for behavioural and emotional problems in children [8], with five subscales: emotional, peer, behavioural and hyperactivity problems and prosocial behaviours. Parent 1 rated their child’s behaviour over the past 6 months on a scale from 0 ‘Not True’ to 2 ‘Certainly True’. The measure has moderate to strong internal reliability and adequate validity in community samples of Australian children [9]. Based on Australian norms [9], children were categorised as having ‘low/no’ mental health symptoms (scoring < 13 out of 40); ‘borderline/query’ mental health symptoms (13–16 out of 40), or ‘abnormal/of concern’ mental health symptoms (≥ 17 out of 40).

*Physical health problems*. Parent 1 reported whether their child had any of the following ongoing conditions: asthma; hearing; vision; recurrent pain (abdominal, headaches, chest, back or other parts of the body); bone, joint or muscle problems; diabetes; epilepsy/seizures; chronic fatigue; or a congenital heart condition. The child’s height and weight were recorded, and body mass index (BMI) calculated. Children were counted as having a physical health problem if their parent reported one of the listed conditions OR the child had overweight or obesity based on the BMI threshold relevant for their age [10].

Other variables included parent education; parent mental illness; arguments in the parents’ relationship; violence in the parents’ relationship; maladaptive parenting (characterised by low warmth and high hostility) by either parent; number of siblings; household income; socioeconomic status; and rurality of home postcode. Full details of all measures are presented in Supplementary Material Table 2.

### Statistical analysis

Analyses were conducted using STATA/SE 16.0 (Statacorp, Texas, US) and the Traj plugin for STATA [11]. Sample characteristics were examined at each wave and presented as means and standard deviations (SD) for continuous variables, and frequencies for categorical variables.

Group-based trajectory modelling (GBTM) was used to identify groups of children that follow qualitatively distinct developmental trajectories of HRQoL, based on children’s scores on the PedsQL. GBTM is a type of finite mixture modelling that uses a maximum likelihood estimator to approximate the unknown distribution of trajectories across population members [12]. GBTM estimates two regression models simultaneously: (1) a multinomial logit model estimating the probability of group-assignment, and (2) models estimating the longitudinal trajectories. Due to the skewed nature of HRQoL data, a censored-normal distribution was used [12]. The group-based trajectory analysis is a maximum likelihood estimator that is reasonable if the missingness is completely at random. However, to increase the precision of the estimated trajectories, children were dropped from analyses if they had fewer than three waves of HRQoL data.

The decision of the optimal number of groups and the functional form of each trajectory was based on the following criteria: [12, 13] (1) higher (i.e. closer to zero) Bayesian information criterion (BIC) and Akaike information criterion (AIC); (2) entropy, which averages the posterior probabilities after individuals have been assigned to their group, where values closer to 1 reflect greater classification accuracy, with a recommended minimum threshold of 0.8^3^; (3) visual inspection of spaghetti plots for how well individual observations clustered around the group mean; (4) whether each group appeared large enough to be clinically meaningful (i.e. > 5% of the population); and (5) whether the groups appeared to be qualitatively distinct (i.e. two groups did not follow a similar trajectory). For the final fitted model, the functional form of each trajectory group was determined by testing combinations of forms (linear, quadratic, cubic or quartic) until the form for each group was statistically significant, and there was no polynomial overfitting (i.e. a linear form modelled as cubic). Additionally, the posterior probabilities (i.e. the probability that an individual belongs to a group) were examined to ensure that the mean posterior probability for each group was greater than 0.7 [12].

A one-group model was first determined (i.e. assuming one underlying population) with additional groups added consecutively. Model fit indices were inspected for 1–6 trajectory group models. All model fit decision metrics and associated output are presented in Supplementary Material Table 3, in line with recommendations for transparency in reporting of trajectory modelling [12]. The same procedures were repeated using the combined sample, and separately for girls and boys.

Clinically important change (a change of more than 4.5 points on the PedsQL^7^) along each trajectory was determined by summarising mean HRQoL scores for each trajectory group at each time point, and calculating the difference between scores at each Wave compared to Wave 1, and compared to each preceding Wave.

### Sensitivity analyses

We conducted the following sensitivity analyses to test the robustness of key decisions made in our trajectory analyses. Firstly, to examine robustness of each child’s trajectory group membership to the shape of each trajectory in the model, we repeated the trajectory analysis using a more parsimonious model with linear forms, in comparison to the more complex forms originally modelled. Trajectory group membership between the linear and more complex models was compared using cross tabulation.

Secondly, we repeated the trajectory modelling using a restricted sample of children with complete HRQoL data for all waves. This enabled a comparison with children whose trajectories are certain, compared to those modelled using three or more waves of data.

## Results

### Sample characteristics

Sample characteristics at each wave are presented in Table 1. Inspection of the sample prior to trajectory modelling indicated that 807 children had fewer than three waves of HRQoL data and were subsequently dropped from analyses. Compared to those who were included in the analyses, in Wave 1, children who were excluded were *more likely* to speak a language other than English (24.7% vs 10.2%); have either parent screen positive for probable serious mental illness (10.8% vs 5.1%, respectively); have parents with an argumentative or violent relationship (29.8 vs 23.5% and 22.8 vs 12.4%, respectively); and were *less likely* to have either parent complete higher education (30.5% vs 51.9%, respectively). In the final trajectory analysis, 4,176 children aged 4–5 years (2,137 boys and 2,039 girls) were included in Wave 1, and 3,071 children (1,568 boys and 1,503 girls; 73.5% of the Wave 1 sample) were retained in the study through to Wave 7, at age 16–17 years.

**Table 1:** Sample characteristics at each wave

	**W1** **4–5 yrs** **(2004)**	**W2** **6–7 yrs** **(2006)**	**W3** **8–9 yrs** **(2008)**	**W4** **10–11 yrs** **(2010)**	**W5** **12–13 yrs** **(2012)**	**W6** **14–15 yrs** **(2014)**	**W7** **16–17 yrs** **(2016)**
Total children, *n*	4,176	4,043	4,084	4,027	3,873	3,497	3,071
**Child characteristics**							
Age*, mean (SD)*	4.7 (0.21)	6.8 (0.24)	8.7 (0.24)	10.8 (0.29)	12.8 (0.31)	14.8 (0.34)	16.9 (0.37)
Sex, male*, n (%)*	2,137 (51.1)	2,066 (51.1)	2,091 (51.2)	2,054 (51.0)	1,978 (51.0)	1,777 (50.8)	1,568 (51.0)
HRQoL (PedsQL)*, mean (SD)*	80.7 (10.4)	80.4 (11.9)	80.1 (12.2)	78.9 (14.2)	81.1 (13.5)	79.4 (15.1)	80.2 (14.1)
Mental health (SDQ)*, mean (SD)*	9.1 (5.2)	7.8 (5.0)	7.5 (5.3)	7.9 (5.5)	7.4 (5.5)	7.1 (5.4)	7.2 (5.5)
Low (< 13), *n (%)*	3,212 (77.0)	3,273 (82.8)	3,105 (84.1)	3,268 (81.6)	3,184 (83.7)	2,843 (84.6)	2,510 (84.0)
Borderline/query (13–16), *n (%)*	554 (13.2)	434 (10.9)	339 (9.1)	415 (10.3)	330 (8.6)	291 (8.6)	242 (8.1)
Abnormal/concern, (17 +), *n (%)*	403 (9.6)	243 (6.1)	246 (6.6)	318 (7.9)	287 (7.5)	225 (6.7)	235 (7.8)
Physical health problem^a^*, n (%)*	1,657 (39.6)	1,584 (39.1)	2,158 (52.8)	2,544 (63.1)	2,415 (62.3)	2,229 (63.7)	2,009 (65.4)
Language other than English, *n (%)*	425 (10.1)	405 (10.0)	407 (9.9)	397 (9.8)	307 (7.9)	337 (9.6)	291 (9.5)
**Parent characteristics**							
Parent 1 sex*,* female,* n (%)*	4,065 (97.3)	3,937 (97.3)	3,976 (97.3)	3,918 (97.2)	3,767 (97.2)	3,402 (97.2)	2,985 (97.2)
Parent 2 sex, male,* n (%)*	3,598 (97.5)	3,496 (97.5)	3,532 (97.5)	3,488 (97.4)	3,359 (97.4)	3,059 (97.4)	2,697 (97.2)
Either parent completed higher education, *n (%)*	2,153 (51.9)	2,088 (52.1)	2,187 (54.4)	2,220 (55.9)	2,217 (57.9)	2,036 (58.6)	1,823 (59.7)
Either parent probable serious mental illness (Kessler 6)*, n (%)*	171 (5.1)	122 (3.7)	139 (4.5)	154 (4.8)	151 (5.1)	127 (4.6)	145 (6.3)
**Family characteristics**							
Either parent maladaptive parenting (low warmth, high hostility)*, n (%)*	446 (12.4)	272 (8.1)	334 (10.6)	426 (13.0)	474 (15.7)	441 (15.7)	293 (13.9)
Argumentative parental relationship, as rated by either parent, *n (%)*	731 (20.3)	577 (16.7)	481 (15.2)	520 (15.7)	502 (16.4)	433 (15.0)	406 (17.0)
Any violence in parental relationship, as rated by either parent, *n (%)*	378 (10.6)	301 (8.9)	261 (8.4)	238 (7.3)	228 (7.7)	202 (7.1)	157 (6.7)
Number of siblings*, mean (SD)*	1.4 (0.9)	1.5 (0.9)	1.6 (1.0)	1.6 (1.0)	1.5 (1.0)	1.4 (1.0)	1.3 (1.0)
Household income*, $AUD, mean (SD)*	77,484.75 (47,386.28)	88,115.54 (62,026.12)	101,858.80 (68,669.39)	110,088.80 (75,791.77)	123,372.60 (86,976.03)	134,479.60 (91,421.19)	149,420.10 (100,911.40)
**Neighbourhood characteristics**							
SEIFA IRSAD^b^*, mean (SD)*	1007.5 (78.4)	1011.2 (73.9)	1012.0 (74.1)	1013.0 (75.0)	1011.6 (73.3)	1013.8 (72.8)	1013.0 (76.1)
Remoteness*,* Major Cities, *n (%)*	2,702 (64.8)	2,616 (64.7)	2,622 (64.3)	2,569 (64.0)	2,458 (63.4)	2,221 (63.5)	1,966 (64.0)

^a^Physical health problems included: asthma; hearing; vision; recurrent pain (abdominal, headaches, chest, back or other parts of the body); bone, joint or muscle problems; diabetes; epilepsy/seizures; chronic fatigue; congenital heart condition; and/or overweight/obese; ^b^ Socio-Economic Index for Areas (SEIFA) Index of Relative Socio-Economic Advantage and Disadvantage (IRSAD)

### Trajectory modelling

**Fig. 1:**
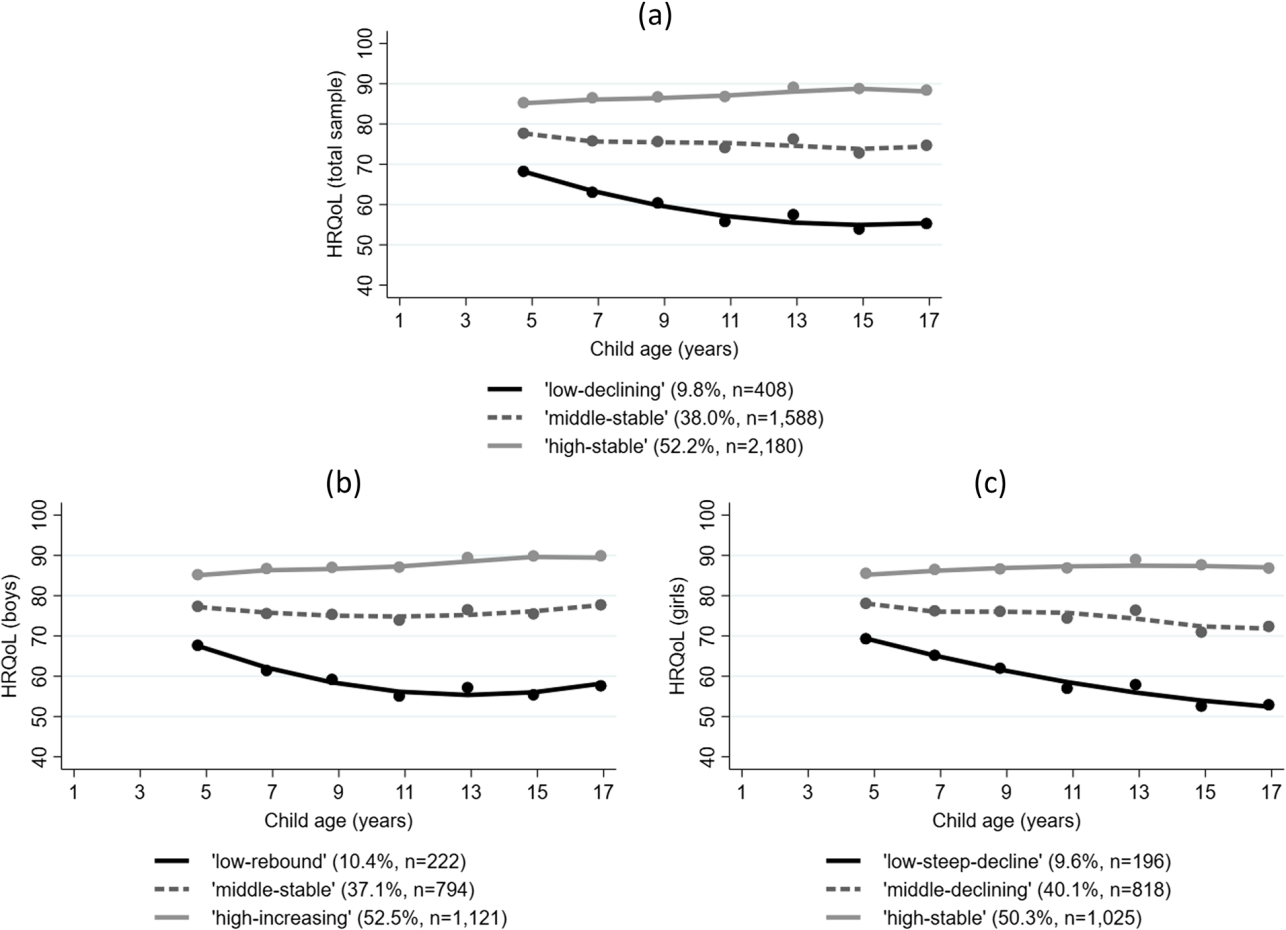
Trajectories of HRQoL for the total sample (**a**), boys (**b**) and girls (**c**). Figure 1 shows the 3-group trajectory models for children aged 4–5 years through to 16–17 years. Trajectories are modelled for the total sample (**a**), and separately for boys (**b**) and girls (**c**). *HRQoL* = health-related quality of life

Based on the model fit criteria described in Sect. 2.3 (see Supplementary Table 3 for decision metric output), a 3-group model was determined to be optimal. The three developmentally distinct trajectories were (Fig. 1): (1) high-stable (52.2% of children); (2) middle-stable (38.0%); and (3) low-declining (9.8%).

When examining trajectories separately by child sex, 3-group models were deemed optimal for both boys and girls (see Supplementary Material Table 3 for decision output), and the proportion of children in each trajectory mirrored that of the total sample model. However, the shape of boys’ and girls’ trajectories showed important differences (Fig. 1). For children in the highest trajectory, girls’ HRQoL scores were stable over time (high-stable), whereas boys saw an *increase* in HRQoL (high-increasing). For children in the middle trajectory, boys’ scores were stable over time (middle-stable), whereas girls’ scores saw declining HRQoL (middle-declining). Both boys and girls in the lowest trajectory saw declining HRQoL, however this was observed as a steeper initial decline for boys with a rebound effect from age 10–11 onwards (low-rebound); yet a steep and continual decline for girls through to age 16–17 (low-steep-decline).

We examined whether the change in HRQoL was clinically important over the period. For children in the high-stable or middle-stable trajectories, mean HRQoL scores did not change significantly between age 4–5 years to 16–17 years (Table 2). However, for children in the improving and declining trajectories, clinically important change was observed. In the total sample, children in the low-declining trajectory had a mean HRQoL score of 68.0 at 4–5 years, with a clinically meaningful decrease in scores at the following wave (mean (M) = 62.7), and a continued decline through to age 16–17 years (M = 54.4). Boys in the high-increasing trajectory saw a clinically meaningful *increase* in HRQoL that was evident by age 14–15 (4–5 years M = 85.3; 14–15 years M = 90.0). Boys in the low-rebound trajectory saw a steep initial decline (4–5 years M = 67.5; 6–7 years M = 61.1), the lowest point at age 10–11 (M = 54.8) and a rebound effect to age 16–17 years (M = 57.2). Girls in the middle-declining trajectory saw a slow decline with a clinically important difference as evident at age 14–15 (4–5 years M = 77.8; 14–15 years M = 70.5). Girls in the low-steep-decline trajectory saw a steep and continual decline from age 4–5 years (M = 69.1) through to 16–17 years (M = 51.8).

**Table 2:**
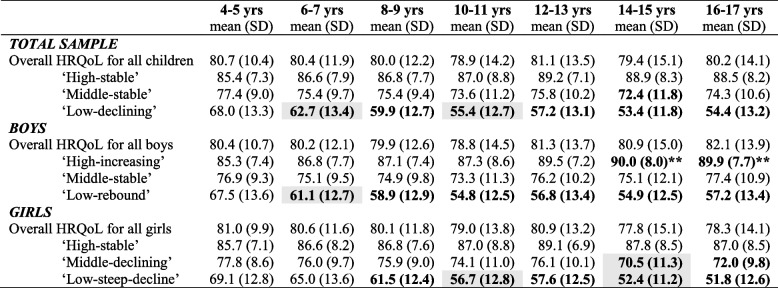
Mean HRQoL and clinically meaningful change at each time point for trajectory groups, overall and by sex

*NB* **Bolding** = a clinically meaningful change (4.5-point change on PedsQL total score) compared to Wave 1; **shading** denotes a clinically meaningful change compared to the previous wave; ** this change is a clinically meaningful increase. HRQoL = Health-related quality of life

In contrast to the distinct trajectories, when examining the sample as a whole, no clinically meaningful change was observed for children’s HRQoL between age 4–5 to 16–17 years for the total sample, or for all boys or all girls together (Table 2). This suggests that the clinically important differences observed for the distinct developmental trajectories are cancelled out at a group or population level.

### Sensitivity analyses

Modelling the trajectories as linear forms largely resulted in children being classified into the same trajectory group as the original analysis: 98.2% of boys (*n* = 2,099/2,137) and 99.3% of girls (*n* = 2,024/2,039), indicating that the results were not dependent on the functional form of the trajectory. No children were reclassified from the highest to lowest HRQoL trajectory groups, or vice versa. (See Supplementary Table 3).

Similarly, in a sensitivity analysis using a sample of children with complete HRQoL data at each wave, trajectory models were not substantially different from the original analysis. Using this sample with more certain trajectories, 91.2% of boys (*n* = 951/1043) and 99.0% of girls (*n* = 1016/1026) were classified into the same trajectory group as the original analysis, and no children were re-classified from the highest to lowest HRQoL trajectory group or vice versa (see Supplementary Table 4).

## Discussion

Our findings reveal that between the ages of 4–17 years, Australian children generally follow three distinct trajectories of HRQoL: approximately half of children have good HRQoL throughout childhood and adolescence, following a high-stable trajectory. The remaining half largely follow a middle-stable trajectory, although one in ten children follow a low-declining trajectory, putting them at risk of continually and increasingly poor HRQoL over time. When examined separately, trajectories for girls and boys show important differences. Boys in the highest trajectory see a clinically meaningful *increase* in their HRQoL by age 14–15 years, and a rebound effect is seen for those in the lowest trajectory after an initial decline. Boys in the middle trajectory have stable HRQoL over time. In contrast, no increasing or rebounding trajectories are observed for girls. While approximately half of girls have high-stable HRQoL, the remaining half have either steadily or rapidly declining HRQoL from age 4–5 to 16–17 years. These findings were robust to sensitivity analyses regarding the shape of the trajectories and sample chosen for the analysis.

Our finding of three distinct trajectories of children’s HRQoL is similar to the two previous trajectory studies in Australian population samples, which reported three [4] or four [3] distinct groups. The number and shape of the trajectories, and the proportion of children in each group in our study mirror those reported by Le et al. [4] The earlier study by Vella et al. [3] used the same sample as our study (followed only to age 12), and found four distinct trajectories, including an overall ‘low-improving’ trajectory that was not identified in our study. However, this trajectory comprised 2% of the population and would have been considered too small in the current study. We did, however, observe the ‘rebounding’ trajectory also reported by Vella et al., though in our study this was only apparent for boys. Previous research has found different trajectory modelling techniques can produce varying results, even when using the same dataset [14]. In light of this, the similarity of our results with previous trajectory analyses using a different dataset (Le et al.) or a different modelling technique (Vella et al.), provides greater confidence in our findings.

This study provides novel evidence of the unique trajectories of HRQoL for girls versus boys throughout childhood and adolescence. For boys, we found stable or increasing/rebounding trajectories, contrasted with only stable or declining trajectories for girls. A similar pattern has been observed in longitudinal studies with one follow-up, where a greater decline was observed for girls at follow-up compared to boys [15–17]. Our findings highlight the importance of considering trajectories of HRQoL separately by sex, as girls are at greater risk of declining HRQoL throughout childhood and adolescence compared to boys.

When considering the sample as a whole, no clinically meaningful change was observed in mean HRQoL scores from age 4–5 years to 16–17 years. However, distinct trajectories of HRQoL were observed over this period, which saw clinically important changes over time. This difference in interpretation of children’s HRQoL at a group level compared to distinct trajectories for boys and girls indicates that considering children’s HRQoL – as a whole – may lead to a false assumption that children’s HRQoL remains stable throughout childhood and adolescence. Our study findings may partially explain the seemingly contradictory findings from previous longitudinal studies that included a baseline and only one follow-up data point. These studies reported a mix of decreasing HRQoL [16, 18–20]; no overall difference [15]; or improvements in HRQoL [21, 22]. The previous studies examined cross-sectional samples of children spanning very large age ranges (e.g. 7–17 years), or at a specific age (e.g. 14–15 years), and followed children over periods ranging from 6 months to 5 years. The previous methodologies employed may have masked any underlying subgroups with the non-linear and contrasting trajectories observed in our study.

Strengths of the study are that we examined a large, national level, longitudinal sample of children spanning childhood and adolescence. We provide the first evidence of the differing trajectories faced by girls and boys over this time. While we believe our study describes a useful set of developmental trajectories of children’s HRQoL, we must still acknowledge that all trajectory analyses are an estimation of ‘true’ trajectories [14]; not all children will follow these trajectories exactly, and the results of our analyses are intended to be a practicable summary of key developmental trajectories at a population level.

The study does have limitations. Ideally we would have used child self-reported HRQoL, however, these data were not available for the duration of the study. Our analysis was conducted separately by sex; while the PedsQL has shown measurement invariance by sex and age and across healthy and chronic health condition groups when self-reported [23–26] this is not known when using parent-report (except see Doostfatemeh et al. [27] for evidence of measurement invariance when using the parent-reported Persian version of the PedsQL). This means we cannot be completely sure our results are not influenced by differences in the way that parents interpret HRQoL for girls/boys, and across different ages. This line of future research is a critical issue, and associated findings would benefit all future studies of children’s HRQoL. Replication of our findings in other countries is warranted, given significant differences have been observed in children’s levels and patterns of HRQoL across countries [5]. Replication could also be conducted using other HRQoL instruments, as our analysis and previous trajectory analyses have all used the PedsQL instrument. Children excluded from the current study due to incomplete data across all waves showed higher levels of disadvantage regarding the risk factors examined at age 4 years, which introduces a level of bias to our results. As these children are likely to have lower HRQoL based on their level of disadvantage, exclusion of these children may mean that the proportion of children following low-declining trajectories may be underestimated in the current study, or that an additional low trajectory is not described.

Our findings are useful for outcome measurement in longitudinal clinical trials, and from a health economic and health policy perspective, as they start to reveal the qualitatively distinct subgroups that may need to be considered individually when assessing the effectiveness of children’s health interventions. Our findings highlight that health policy decision-making should not consider ‘children’s HRQoL’ a static variable, but rather a dynamic variable that changes over time, and by sex, and which comprises numerous qualitatively distinct subgroups. The presence of these distinct trajectories of children’s HRQoL should be taken into consideration when assessing the effectiveness of treatments and interventions that may impact HRQoL throughout childhood and adolescence. Failure to account for these pre-existing trajectories may over- or under-estimate treatment effects, by attributing change in HRQoL to treatments where an increase or decrease in HRQoL was expected regardless of treatment. Our results thereby help to ensure that healthcare policy and financing decision-making that relies on assessment of the effectiveness and cost-effectiveness of treatments and interventions could be strengthened by the consideration of the qualitatively distinct developmental trajectories of HRQoL identified in this study.

## Conclusions

Our results are the first to describe developmentally distinct trajectories of HRQoL faced by boys and girls throughout childhood and adolescence. We found approximately half of Australian children have good HRQoL throughout childhood and adolescence, though one in ten children follow a low and declining trajectory over time. Important differences in the trajectories of boys and girls saw increasing and rebounding trajectories for boys, yet only steady or declining trajectories for girls. These qualitatively distinct developmental trajectories of HRQoL should be considered when assessing the effectiveness of treatments and interventions that may impact HRQoL, so as to not over- or under-estimate treatment effects in healthcare policy and financing decision-making.

## Supplementary Information

**Additional file 1.**
