# Supplementary material for: Distinct developmental trajectories of health-related quality of life for boys and girls throughout childhood and adolescence; a national level longitudinal study
Source: Health Qual Life Outcomes. 2023 Aug 1;21:82. doi: 10.1186/s12955-023-02171-5 (PMC10394779; doi:10.1186/s12955-023-02171-5)
Supplement: Supplementary file 1 — Additional file 1. [file 12955_2023_2171_MOESM1_ESM.docx]

**SUPPLEMENTARY MATERIAL**

**Table of Contents**

Table 1: Summary of PedsQL HRQoL instrument items and domains ……………….….……………………….pg. 2

Table 2: Full details of variables included in study ……………………………………………………………….pg. 3

Table 3: Decision metrics and associated data output for group-based trajectory modelling …………………….pg. 6

Detailed decision metrics ………………………………………………………………………………...pg. 6

ALL sample output (BIC, AIC, Entropy, trajectory figures, spaghetti plots) …………………………...pg. 7

ALL sample output (shape modelling for 3-group model, posterior probabilities) ….……………….....pg. 8

ALL sample output (*Sensitivity analysis*: trajectory shape, linear; cross tab with original model) ……..pg. 9

BOYS output (BIC, AIC, Entropy, trajectory figures, spaghetti plots) ……….……………..................pg. 10

BOYS output (shape modelling for 3-group model, posterior probabilities) ……….………………….pg. 11

BOYS output (*Sensitivity analysis*: trajectory shape, linear; cross tab with original model) …………..pg. 12

GIRLS output (BIC, AIC, Entropy, trajectory figures, spaghetti plots) ………………………………..pg. 13

GIRLS output (shape modelling for 3-group model, posterior probabilities) ……….…………………pg. 14

GIRLS output (*Sensitivity analysis*: trajectory shape, linear; cross tab with original model)…………..pg. 15

Table 4: *Sensitivity analysis*: trajectory modelling with complete PedsQL data ………………………..……….pg. 16

References ………………………………………………………………………………………………………..pg. 22

**TABLE 1. Summary of PedsQL HRQoL instrument items and domains.**

| **Domain** | **Item** | **Response options** |
| --- | --- | --- |
| *Header question for instrument*: | “In the past one month how often would you say this child has had a problem with…” | 1 Never  2 Almost never  3 Sometimes  4 Often  5 Almost always |
| Physical functioning (8 items) | 1. Walking more than one block | |
|  | 1. Running | |
|  | 1. Participating in sports activity or exercise | |
|  | 1. Lifting something heavy | |
|  | 1. Taking a bath or shower by him/herself | |
|  | 1. Doing chores like picking up his/her toys | |
|  | 1. Having hurts or aches | |
|  | 1. Having a low energy level / tired | |
| Emotional functioning (5 items) | 1. Feeling afraid or scared | |
|  | 1. Feeling sad or blue | |
|  | 1. Feeling angry | |
|  | 1. Trouble sleeping | |
|  | 1. Worrying about what will happen to him/her | |
| Social functioning (5 items) | 1. Getting along with other children | |
|  | 1. Other children not wanting to be his/her friend | |
|  | 1. Getting teased by other children | |
|  | 1. Not being able to do things that other children his/her age can do | |
|  | 1. Keeping up when playing with other children | |
| School functioning (5 items) | 1. Paying attention in class | |
|  | 1. Forgetting things | |
|  | 1. Keeping up with school activities | |
|  | 1. Missing school because of not feeling well | |
|  | 1. Missing school to go to the doctor or hospital | |

**NOTE**: This item list reflects the **PedsQL parent-report version for children aged 8-12 years**. The PedsQL has different age-appropriate versions available by parent-report for ages 2-4, 5-7, 8-12 and 13-18. There are minor variations in wording on some items according to age.

**TABLE 2. Full details of variables included in study**

| **Construct** | **Data relate to:**  **(NB: this is not the respondent)** | | | | **Description** |
| --- | --- | --- | --- | --- | --- |
|  | **SC** | **P1** | **P2** | **H** |  |
| *Child age* | X |  |  |  | Recorded at each wave based on the child’s birth date. |
| *Child sex* | X |  |  |  | Reported by Parent 1 at Wave 1, and this value was continued to other waves. Coded as 0 = ‘male’ and 1 = ‘female’. |
| *Language other than English (LOTE)* | X |  |  |  | Measured as the main language spoken by the child at home. Dichotomised and coded as 0 = ‘English’; 1 = ‘Language other than English (LOTE)’. |
| *Health-related quality of life (HRQoL)* | X |  |  |  | At each wave, children’s health-related quality of life (HRQoL) was measured using the Pediatric Quality of Life Inventory (PedsQL), a 23-item generic measure of HRQoL comprising four domains: physical; emotional; social; and school functioning.^3^ The PedsQL is feasible, valid and reliable in general population research.^3^ Parent 1 rated their child’s functioning over the last month on a scale from 1 ‘never a problem’ to 5 ‘almost always a problem’. Items were reverse scored and linearly transformed to a 0–100 scale such that higher scores represent better HRQoL. A total score was calculated as the sum of each item divided by the number of items answered at each time point, provided less than half of the items were missing.^3^ A 4.5 point change in the PedsQL total score is considered a clinically meaningful difference using the parent proxy-report form.^3^ |
| *Mental health problem* | X |  |  |  | Children’s mental health symptoms were measured using the Strengths and Difficulties Questionnaire (SDQ), a widely used screening instrument for assessing behavioural and emotional problems in children,^4^ with five subscales: emotional, peer, behavioural and hyperactivity problems and prosocial behaviours. Parent 1 rated their child’s behaviour over the past 6 months on a scale from 0 ‘Not True’ to 2 ‘Certainly True’. The measure has moderate to strong internal reliability and adequate validity in community samples of Australian children.^5,6^ Higher scores reflect greater symptoms/problems. Based on Australian norms,^6^ a total score of 13-16 (out of 40) indicates ‘borderline/query’ symptoms, and scores ≥17 indicate ‘abnormal/of concern’ mental health symptoms. |
| *Physical health problem* | X |  |  |  | Parent 1 reported whether their child had any ‘ongoing condition(s)’; defined in LSAC as conditions that exist for some period of time – weeks, months or years – or re-occur regularly; they do not have to be diagnosed by a doctor. Conditions included here were: asthma; hearing; vision; recurrent pain (abdominal, headaches, chest, back or other parts of the body); bone, joint or muscle problems; diabetes; epilepsy/seizures; chronic fatigue; and/or congenital heart condition.  Additionally, the child’s height and weight were recorded, and BMI calculated. Children were counted as having a physical health problem if their weight status was overweight or obese based on the BMI threshold relevant for their age (see Cole et al.^7^). |
| *Parent sex* |  | X | X |  | Parent 1 and Parent 2 self-reported their sex at Wave 1 and this value was continued to other waves. Coded as 0 = ‘male’ and 1 = ‘female’. |
| *Parent education* |  | X | X |  | Measured as Parent 1 and Parent 2 self-reported completion of university level higher education, including postgraduate degree; graduate diploma / certificate; bachelor degree; advanced diploma / diploma. Binary variable coded as 0 = ‘no’ (if both parents had not completed higher education); and 1 = ‘yes’ (if at least one parent had completed higher education). If the child had only one parent in the household, this variable reflects the responses for Parent 1 only. |
| *Parent mental illness* |  | X | X |  | Measured for both Parent 1 and Parent 2 using the Kessler 6 screening scale;^8^ a six item, validated screening scale that asks “In the past four weeks, about how often did you feel… nervous”, “hopeless”, “restless or fidgety”, “that everything was an effort”, “so sad that nothing could cheer you up”, and “worthless”, scored from 1 ‘all the time’ to 5 ‘none of the time’. Scores were reverse coded such that higher scores reflect greater problems. Total scores were dichotomised in line with the recommended clinical cut-points of 0 = ‘Score of 6-18, No probable serious mental illness’; 1 = ‘Score of ≥19, Probable serious mental illness’. In the current study, ‘*parent mental illness*’ was indicated if at least one parent screened positive for mental health problems. If the child had only one parent in the household, this variable reflects the responses for Parent 1 only. |
| *Maladaptive parenting* |  | X | X |  | In line with previous research,^10,11^ we used a composite measure to indicate *maladaptive parenting*, combining low *warmth* and high *hostility* in parenting behaviours. *Parental warmth* was measured as the mean of six items, e.g. “How often do you express affection by hugging, kissing and holding this child”. *Parental hostility* was measured as the mean of six items, e.g. “How often do you tell this child that he/she is bad or not as good as others”. Both were rated on a 5-point scale from 1 ‘never/almost never’ to 5 ‘all the time’, such that higher scores represent higher levels of parenting *warmth* and *hostility*. Mean scores were divided into quintiles, and *maladaptive parenting* was indicated if at least one parent scored in the highest quintile for *hostility* AND the lowest quintile for *warmth*. If the child had only one parent in the household, this variable reflects the responses for Parent 1 only. |
| *Argumentative relationship* |  | X | X |  | An argumentative parent relationship was measured as the mean of four items, completed separately by Parent 1 and Parent 2. Parents responded to the questions: How often… “do you and your partner disagree about basic child-rearing issues?”; “is your conversation awkward or stressful?”; “do you argue?”; “is there anger or hostility between you?”, with responses rated from 1 ‘never’ to 5 ‘always’. The mean score was taken for each Parent, and argumentative relationship was indicated (coded as ‘1’) if the mean score from either Parent was ≥3 (i.e. at least ‘sometimes’. This cut point was chosen to select only the more severe end based on the histogram of responses. This variable was coded s ‘0’ when both Parents’ mean score was <3. Single parents were rated as ‘0’ due to not being in a relationship with someone living in the household. |
| *Violence in relationship* |  | X | X |  | Violence in the parents’ relationship was measured with a single item, completed separately by Parent 1 and Parent 2. Parents responded to the question: “How often do you have arguments with your partner that end up with people pushing, hitting, kicking or shoving?", with responses rated from 1 ‘never’ to 5 ‘always’. The reported score was taken for each Parent, and violence in the relationship was indicated (coded as ‘1’) if the score from either Parent was ≥2 (i.e. at least ‘rarely’). This cut point was chosen to select only the more severe end based on the histogram of responses. This variable was coded s ‘0’ when both Parents’ scores were ‘1’ (never). Single parents were rated as ‘0’ due to not being in a relationship with someone living in the household. |
| *Number of siblings* |  |  |  | X | Parent 1 reported at each Wave the total number of children in the household (other than the study child). Dichotomised and coded as 0 = ‘0 or 1 sibling(s)’; 1 = ‘2 or more siblings’. |
| *Annual household income* |  |  |  | X | Parents reported household income at each wave as the combined income of all adults in the household. Where possible, missing income values were imputed by the LSAC team; the imputation methods employed are described in detail elsewhere.^15^ Values were categorised based on the 2021 Australian Taxation Office income brackets, and coded as 1 = ‘$0-$18,200’; 2 = ‘$18,201-$45,000’; 3 = ‘$45,001-$120,000’; 4 = ‘$120,001-$180,000’; 5 = ‘$180,001 and over’. |
| *Socioeconomic status* |  |  |  | X | Socioeconomic status was estimated using the Socio-Economic Index for Areas (SEIFA) score, specifically, the Index of Relative Socio-Economic Advantage and Disadvantage (IRSAD).^16^ This score is assigned for every Australian postcode based on census data reflecting the economic and social conditions of people and households within an area. It is designed to have a national mean of 1,000 and SD of 100, where a lower score represents greater disadvantage and a higher score represents greater advantage. SEIFA IRSAD scores were classified into quintiles for use in analyses. |
| *Rurality* |  |  |  | X | Rurality of the child’s home postcode was dichotomised as 1 = ‘Major Cities of Australia’ and 0 = ‘Other’, which included all other categories (i.e. regional, rural etc.) in line with the Australian Statistical Geography Standard levels of remoteness.^17^ |

*SC* = study child; *P1* = Parent 1 (the child’s primary caregiver); *P2* = Parent 2 (the primary caregiver’s partner, if applicable); *H* = household.

**TABLE 3. Decision metrics and associated data output for group-based trajectory modelling.**

**3.1 Detailed decision metrics (copy of text within main manuscript)**

The decision of the optimal number of groups and the functional form of each trajectory was based on the following criteria:^19,20^ (1) higher (i.e. closer to zero) Bayesian information criterion (BIC) and Akaike information criterion (AIC); (2) entropy, which averages the posterior probabilities after individuals have been assigned to their group, where values closer to 1 reflect greater classification accuracy, with a recommended minimum threshold of 0.8^21^; (3) visual inspection of spaghetti plots for how well individual observations clustered around the group mean; (4) whether each group appeared large enough to be clinically meaningful (i.e. >5% of the population); and (5) whether the groups appeared to be qualitatively distinct (i.e. two groups did not follow a similar trajectory). For the final fitted model, the functional form of each trajectory group was determined by testing combinations of forms (linear, quadratic, cubic or quartic) until the form for each group was statistically significant, and there was no polynomial overfitting (i.e. a linear form modelled as cubic). Additionally, the posterior probabilities (i.e. the probability that an individual belongs to a group) were examined to ensure that the mean posterior probability for each group was greater than 0.7.^19^

**3.2 Trajectory modelling output**

The pages below provide output for the decision metrics listed above. Firstly, for modelling trajectories for the total sample; followed by the sample of boys, then the sample of girls.

**3.2.1 ALL SAMPLE OUTPUT**

**Decision statistics for the number and shape of trajectory groups – ALL SAMPLE**

| **HRQOL** | % of population in each group | BIC | AIC | Entropy |
| --- | --- | --- | --- | --- |
| 1 group, quartic | 100% | -98649.52 | -98630.51 | ---- |
| 2 groups, quartic | 30%; 70% | -95163.18 | -95125.16 | 0.842 |
| 3 groups, quartic | 10%; 38%; 52% | -94133.16 | -94076.13 | 0.807 |
| 4 groups, quartic | 5%; 19%; 40%; 36% | -93859.88 | -93783.83 | 0.740 |
| 5 groups, quartic | 2%; 10%; 27%; 41%; 20% | -93729.79 | -93634.73 | 0.721 |
| 6 groups, quartic | 2%; 9%; 9%; 19%; 41%; 21% | -93549.92 | -93435.85 | 0.714 |

**Trajectory figures for each model**


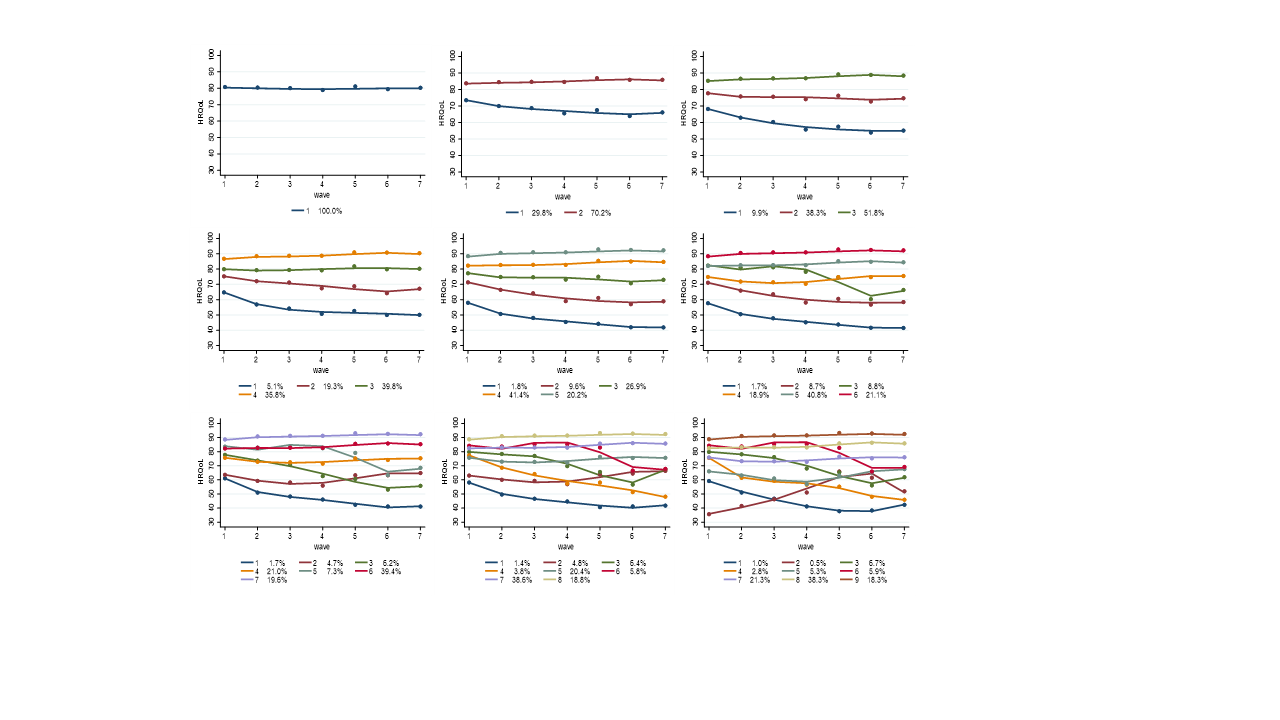


**Spaghetti plots for each model**
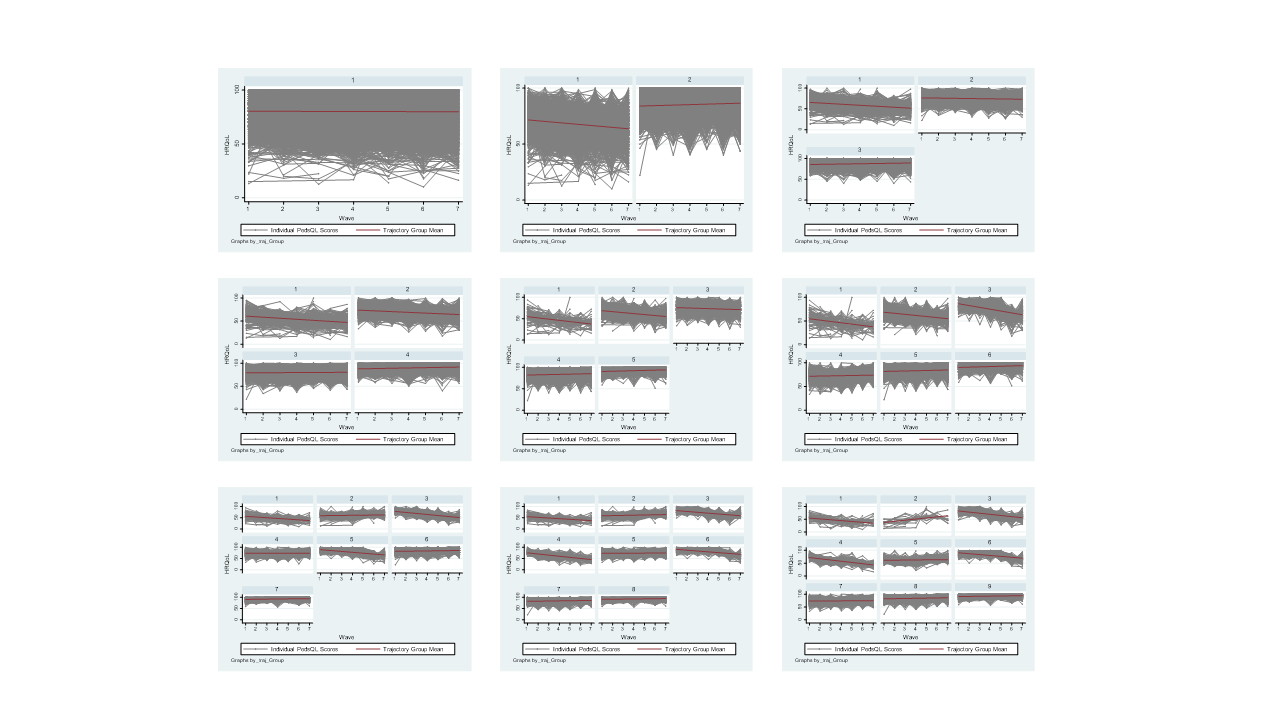


**Decision – 3-group model:**

- Sufficient entropy (>.80), BIC, AIC (rejected 4^th^ traj model because of this)
- Qualitatively distinct trajectories (rejected 4^th^ traj model because of this)
- Large enough groups (>5%; rejected 5^th^ and 6^th^ traj model because of this)
- Spaghetti plots cluster around mean

**Final 3-group model: Shape of trajectory groups – ALL SAMPLE**


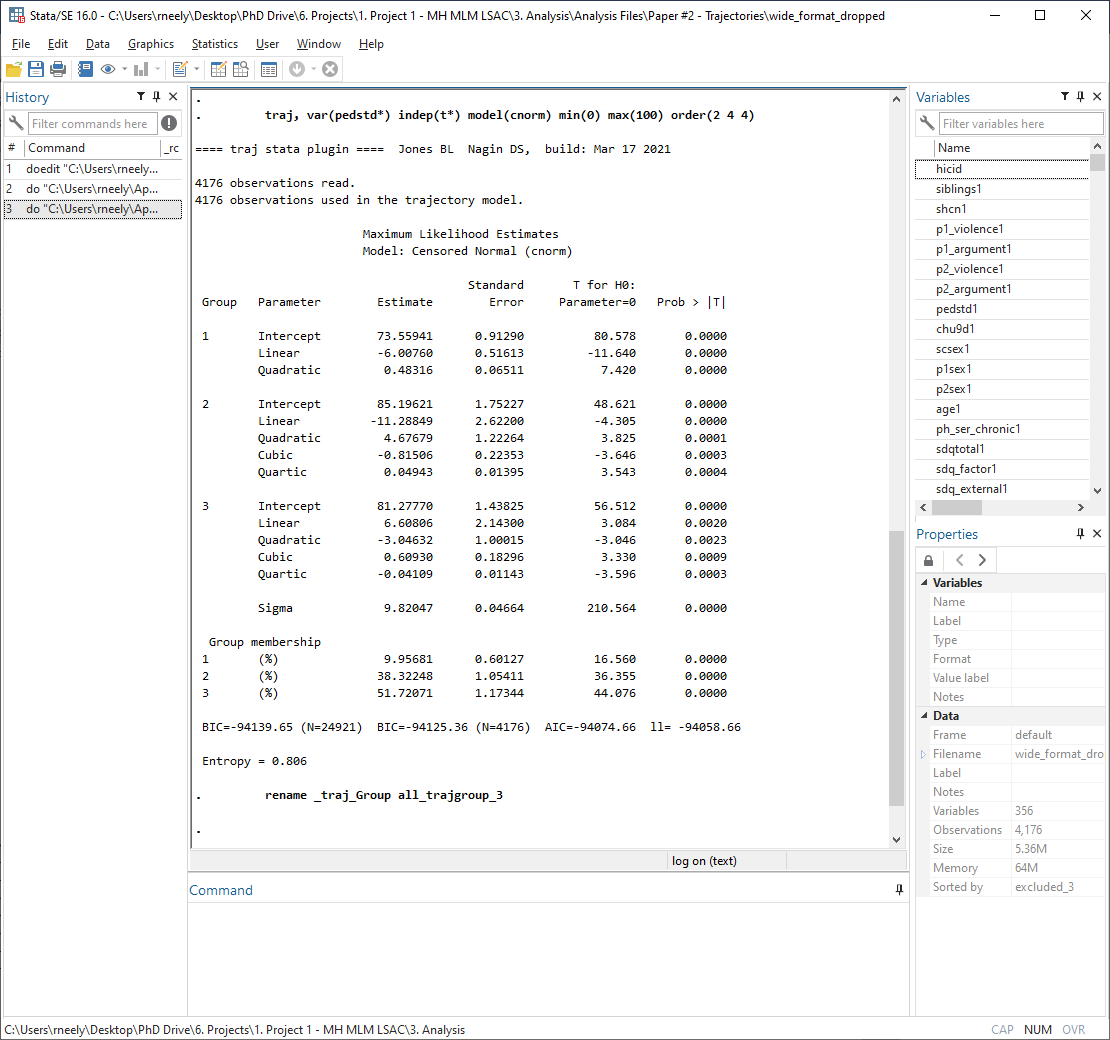

**Final 3-group model: posterior probabilities (PP) of individual membership within each group**

| **Trajectory Group – FOR ALL SAMPLE** | Obs. | Mean PP | SD | Min. | Max. |
| --- | --- | --- | --- | --- | --- |
| Group 1 | 408 | 0.91 | 0.13 | 0.50 | 1.00 |
| Group 2 | 1,588 | 0.88 | 0.14 | 0.50 | 0.99 |
| Group 3 | 2,180 | 0.92 | 0.12 | 0.50 | 1.00 |

**Sensitivity Analysis on shape of trajectories – ALL SAMPLE**

**Modelling all as linear as sensitivity analysis**


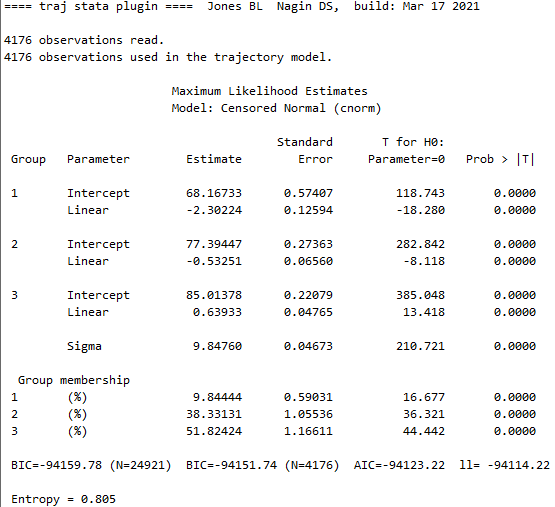

**Sensitivity 3-group model: posterior probabilities (PP) of individual membership within each group**

| **Trajectory Group – FOR ALL SAMPLE** | Obs. | Mean PP | SD | Min. | Max. |
| --- | --- | --- | --- | --- | --- |
| Group 1 | 400 | 0.92 | 0.12 | 0.50 | 1.00 |
| Group 2 | 1,597 | 0.88 | 0.14 | 0.50 | 0.99 |
| Group 3 | 2,179 | 0.93 | 0.12 | 0.50 | 1.00 |

**Compare trajectory group membership between original modelled form and all linear form**


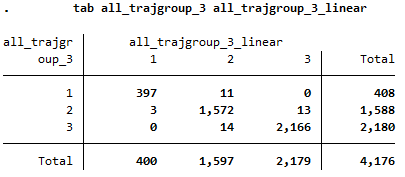


**3.2.2 BOYS OUTPUT**

**Decision statistics for the number and shape of trajectory groups – BOYS**

| **BOYS HRQOL** | % of population in each group | BIC | AIC | Entropy |
| --- | --- | --- | --- | --- |
| 1 group, quartic | 100% | -50517.35 | -50500.35 | ---- |
| 2 groups, quartic | 26%; 73% | -48613.39 | -48579.38 | 0.874 |
| 3 groups, quartic | 10%; 37%; 52% | -48090.93 | -48039.93 | 0.805 |
| 4 groups, quartic | 6%; 19%; 41%; 34% | -47939.94 | -47871.93 | 0.745 |
| 5 groups, quartic | 2%; 8%; 21%; 41%; 28% | -47889.04 | -47804.03 | 0.729 |
| 6 groups, quartic | 2%; 8%; 9%; 14%; 41%; 27% | -47798.10 | -47696.09 | 0.723 |

**Trajectory figures for each model**
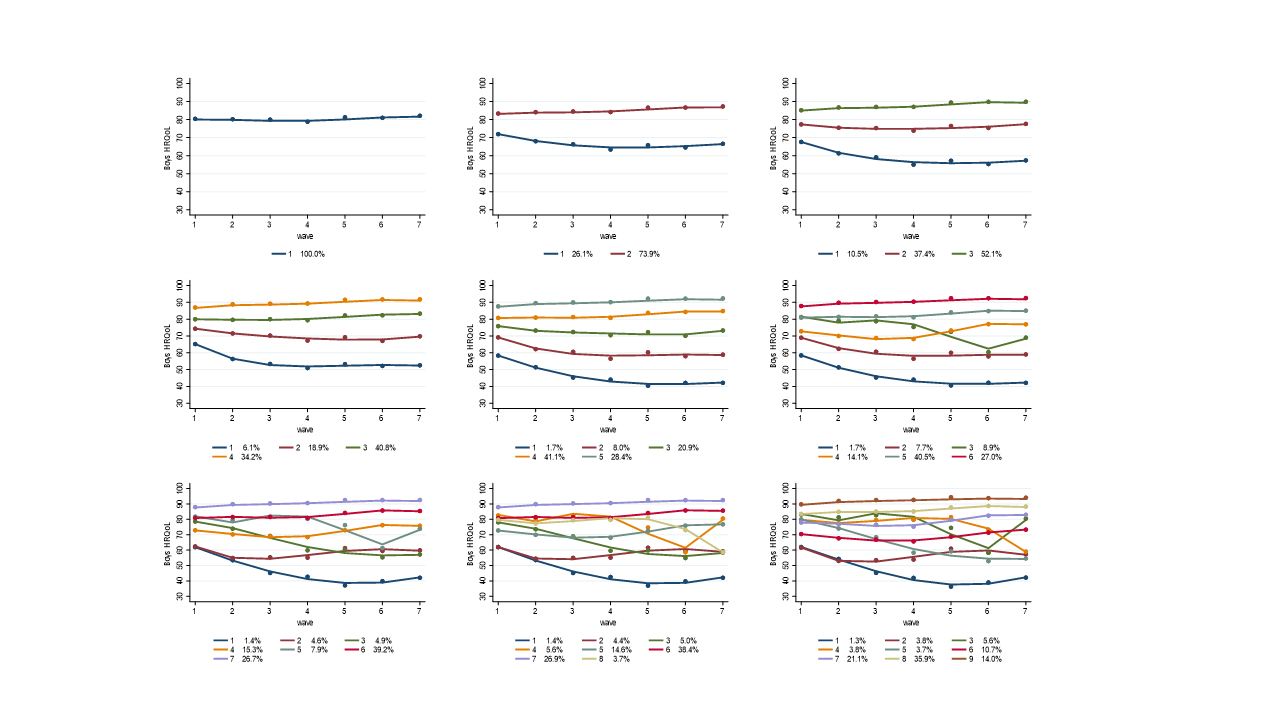


**Spaghetti plots for each model**


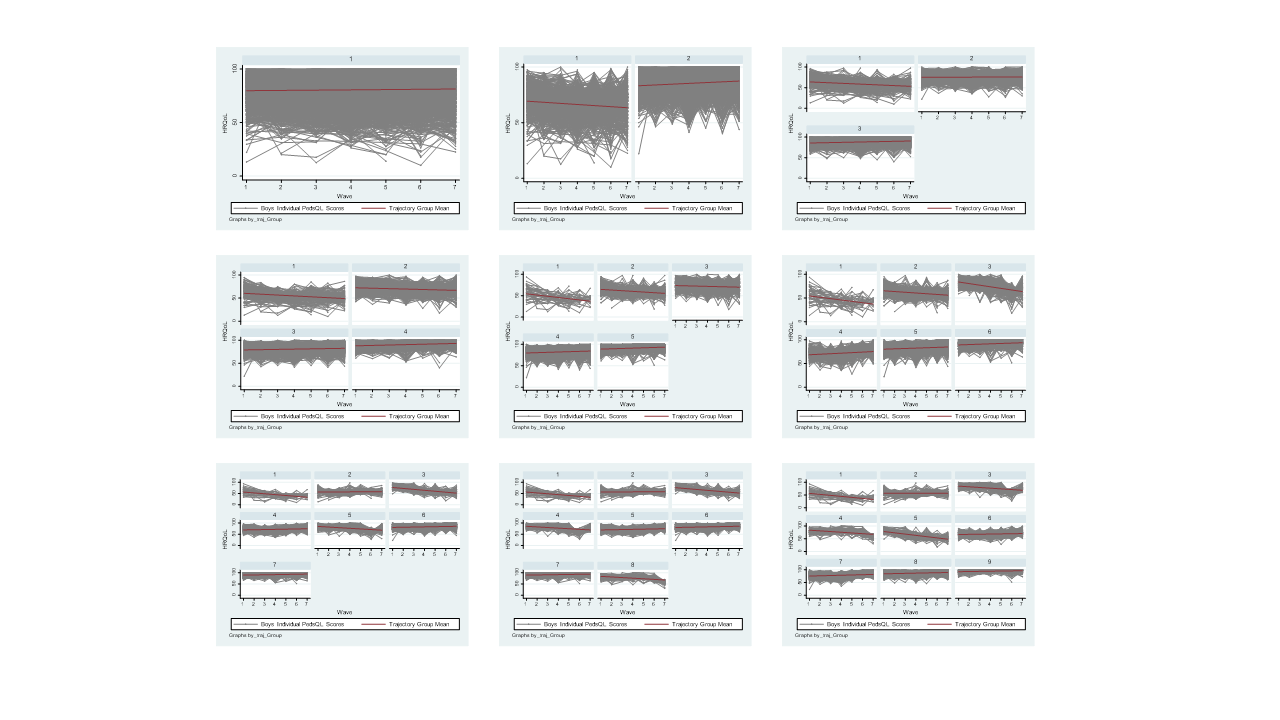


**Decision – 3-group model:**

- Sufficient entropy (>80), BIC, AIC (rejected 4^th^ traj model because of this)
- Qualitatively distinct trajectories (rejected 4^th^ traj model because of this)
- Large enough groups (>5%; rejected 5^th^ and 6^th^ traj model because of this)
- Spaghetti plots cluster around mean

**Final 3-group model: Shape of trajectory groups – FOR BOYS**


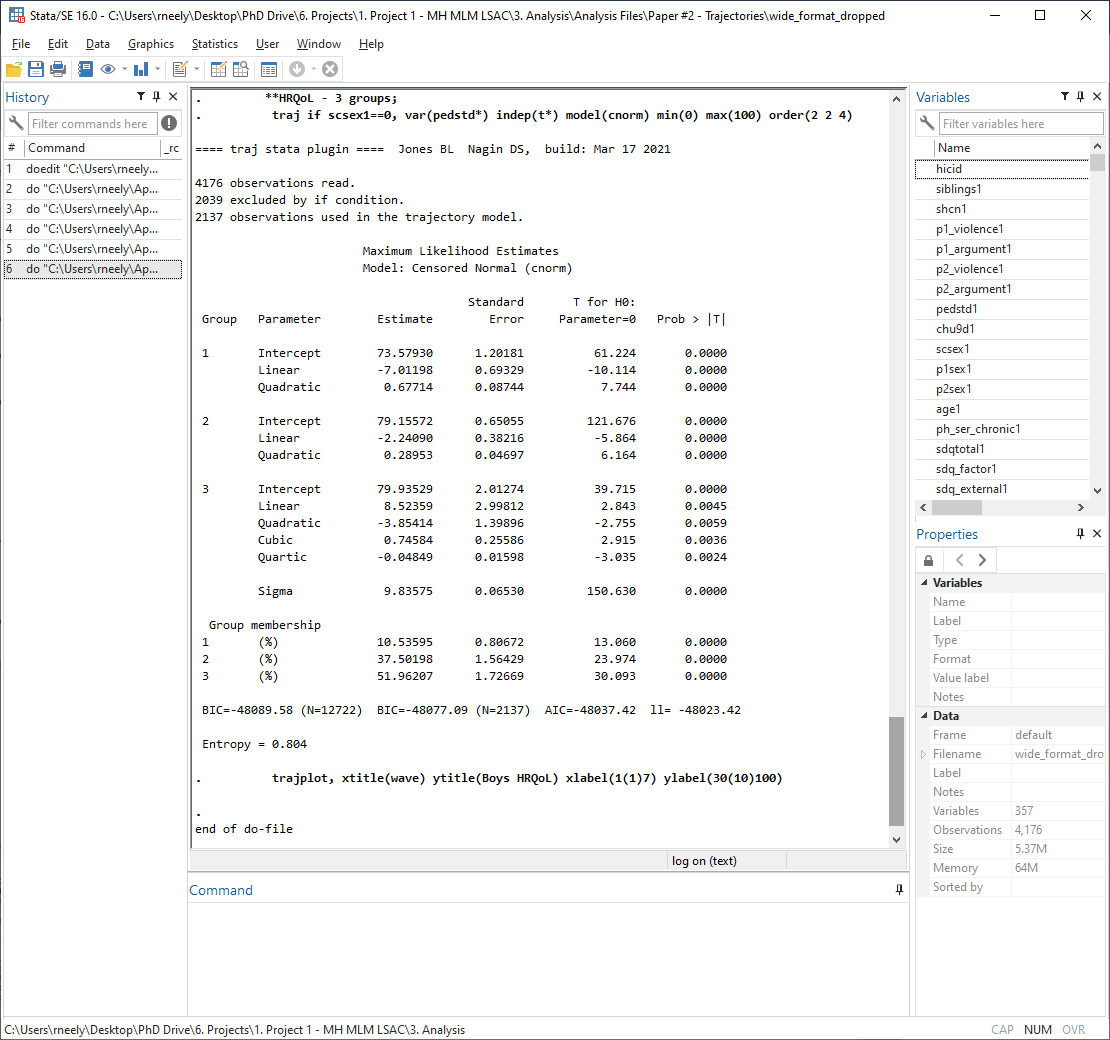

**Final 3-group model: posterior probabilities (PP) of individual membership within each group**

| **Trajectory Group – FOR BOYS** | Obs. | Mean PP | SD | Min. | Max. |
| --- | --- | --- | --- | --- | --- |
| Group 1 | 222 | 0.93 | 0.12 | 0.50 | 1.00 |
| Group 2 | 794 | 0.87 | 0.14 | 0.50 | 0.99 |
| Group 3 | 1,121 | 0.92 | 0.12 | 0.50 | 1.00 |

**Sensitivity Analysis on shape of trajectories – BOYS**


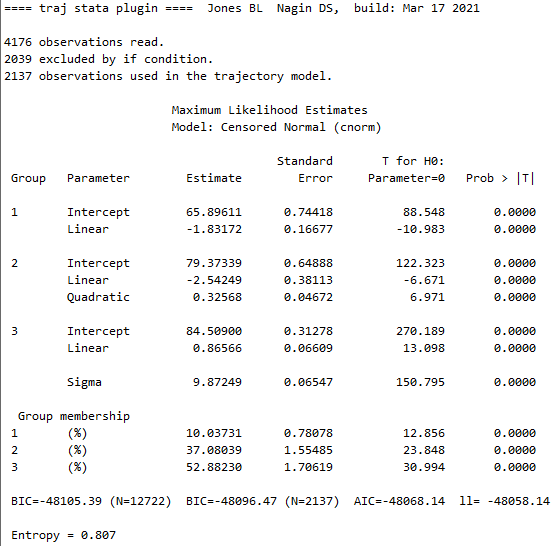
**Modelling all closer to linear as sensitivity analysis**

**Sensitivity 3-group model: posterior probabilities (PP) of individual membership within each group**

| **Trajectory Group – FOR BOYS** | Obs. | Mean PP | SD | Min. | Max. |
| --- | --- | --- | --- | --- | --- |
| Group 1 | 209 | 0.94 | 0.12 | 0.52 | 1.00 |
| Group 2 | 790 | 0.88 | 0.15 | 0.50 | 0.99 |
| Group 3 | 1138 | 0.92 | 0.12 | 0.50 | 1.00 |

**Compare trajectory group membership between original modelled form and more linear form**


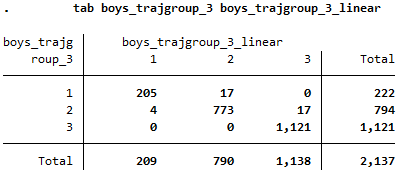


**3.2.3 GIRLS OUTPUT**

**Decision statistics for the number and shape of trajectory groups – GIRLS**

| **GIRLS HRQOL** | % of population in each group | BIC | AIC | Entropy |
| --- | --- | --- | --- | --- |
| 1 group, quartic | 100% | -48091.51 | -48074.65 | ---- |
| 2 groups, quartic | 34%; 66% | -46487.33 | -46453.61 | 0.812 |
| 3 groups, quartic | 10%; 40%; 50% | -45994.35 | -45943.76 | 0.810 |
| 4 groups, quartic | 3%; 14%; 39%; 44% | -45878.18 | -45810.74 | 0.794 |
| 5 groups, quartic | 2%; 10%; 31%; 41%; 15% | -45819.05 | -45734.75 | 0.738 |
| 6 groups, quartic | 2%; 9%; 24%; 40%; 8%; 16% | -45756.70 | -45655.54 | 0.721 |

**Trajectory figures for each model**
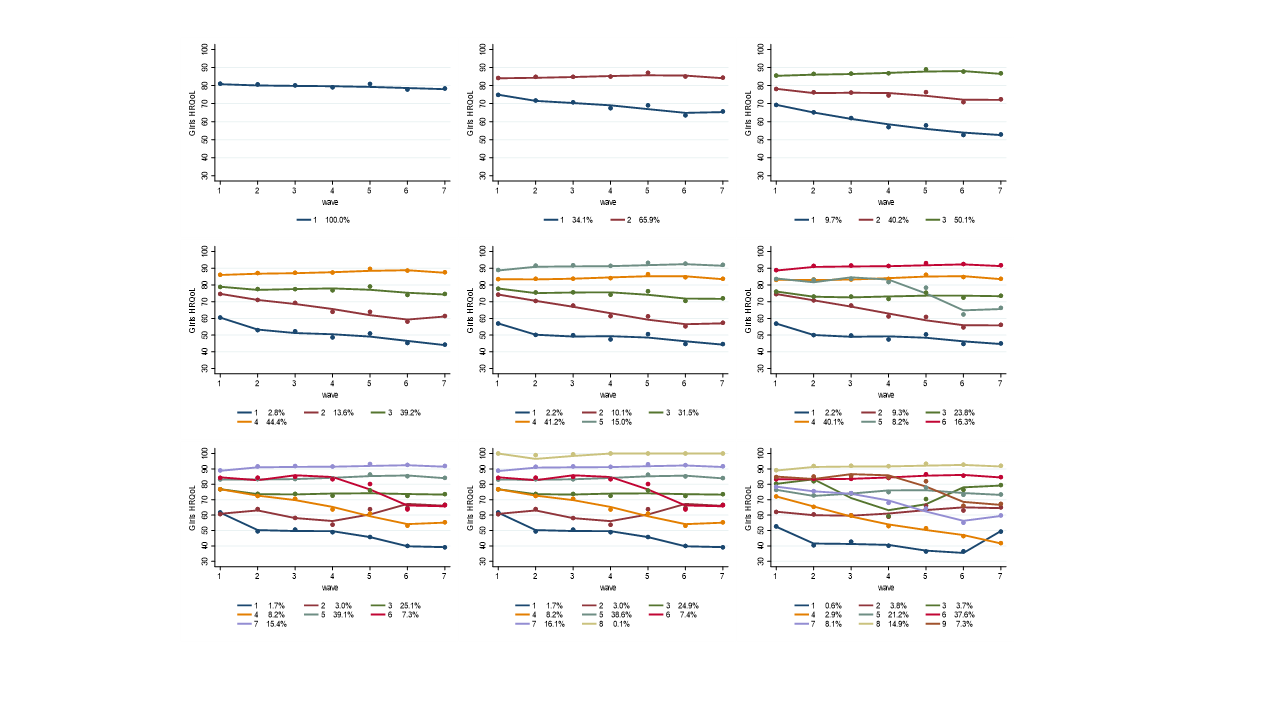


**Spaghetti plots for each model**
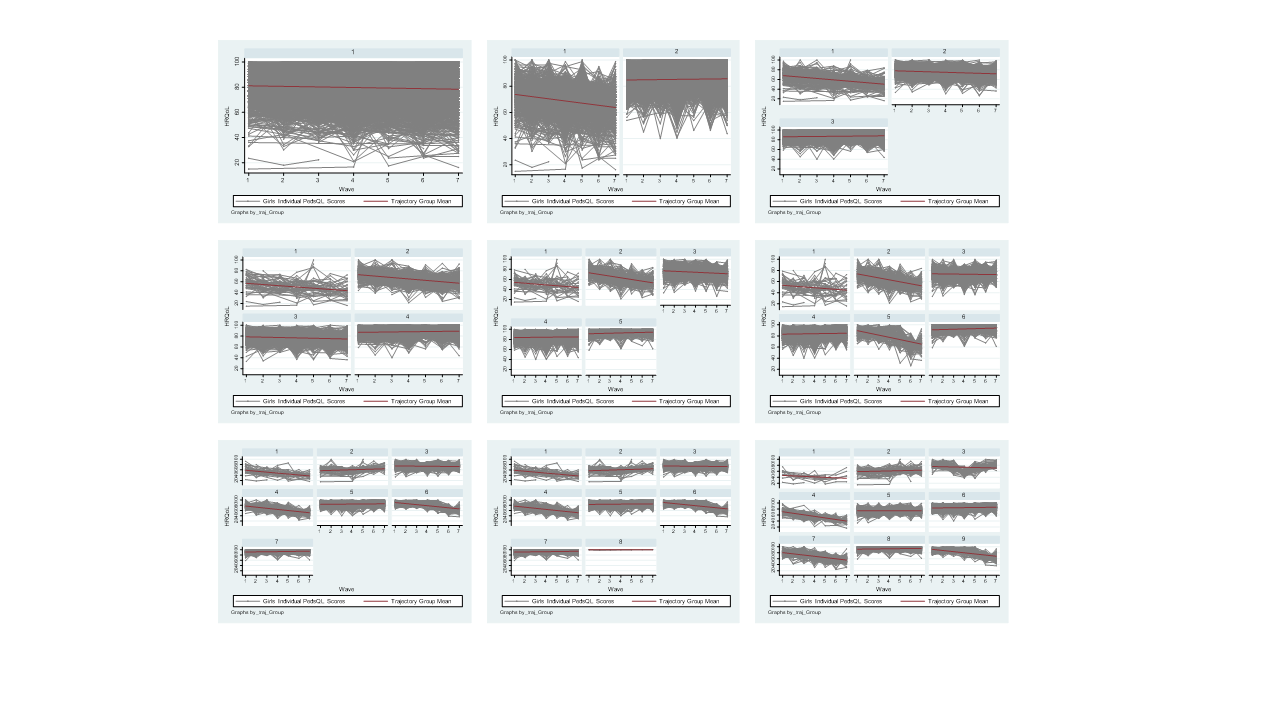


**Decision – 3-group model:**

- Sufficient entropy (>80), BIC, AIC (rejected 4^th^ traj model because of this)
- Qualitatively distinct trajectories (rejected 4^th^ traj model because of this)
- Large enough groups (>5%; rejected 4^th^ 5^th^ 6^th^ traj model because of this)
- Spaghetti plots cluster around mean

**Final 3-group model: Shape of trajectory groups – FOR GIRLS**


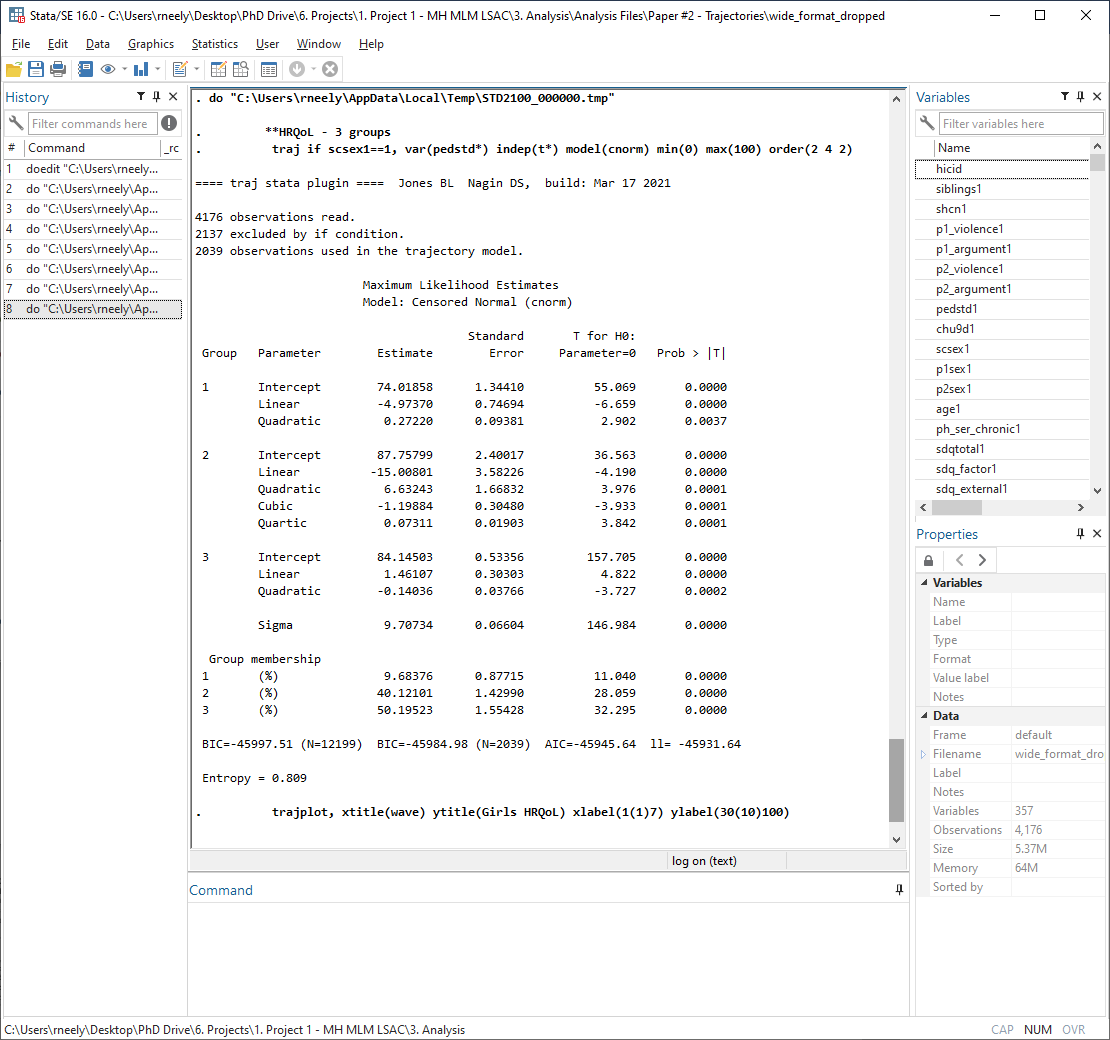

**Final 3-group model: posterior probabilities (PP) of individual membership within each group**

| **Trajectory Group – FOR GIRLS** | Obs. | Mean PP | SD | Min. | Max. |
| --- | --- | --- | --- | --- | --- |
| Group 1 | 196 | 0.90 | 0.13 | 0.51 | 1.00 |
| Group 2 | 818 | 0.88 | 0.13 | 0.50 | 0.99 |
| Group 3 | 1,025 | 0.93 | 0.11 | 0.50 | 1.00 |

**Sensitivity Analysis on shape of trajectories – GIRLS**

**Modelling all as linear for sensitivity analysis**


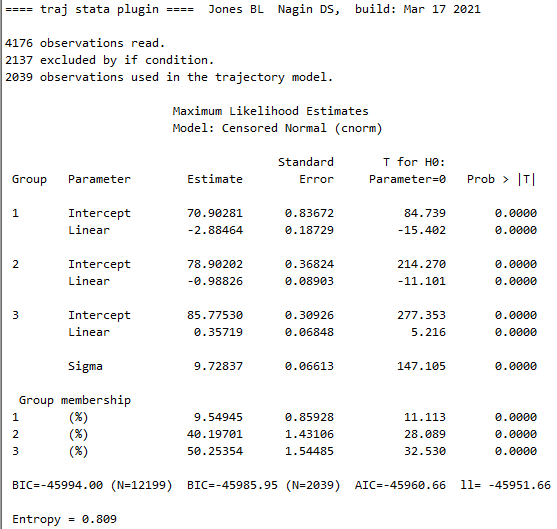

**Sensitivity 3-group model: posterior probabilities (PP) of individual membership within each group**

| **Trajectory Group – FOR GIRLS** | Obs. | Mean PP | SD | Min. | Max. |
| --- | --- | --- | --- | --- | --- |
| Group 1 | 193 | 0.91 | 0.13 | 0.52 | 1.00 |
| Group 2 | 817 | 0.89 | 0.14 | 0.51 | 0.99 |
| Group 3 | 1029 | 0.93 | 0.12 | 0.50 | 1.00 |

**Compare trajectory group membership between original modelled form and more linear form**


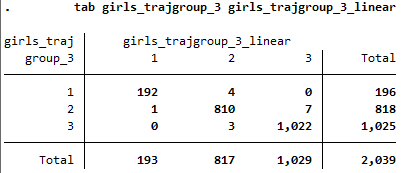


**TABLE 4: Sensitivity analysis on sample chosen for trajectory modelling**

NOTE: The below analyses fully replicate the main trajectory modelling, using a more restricted sample of children – only those with complete PedsQL data for all waves.

**4.1 SENSITIVITY ANALYSIS: ALL SAMPLE**

**Decision statistics for the number and shape of trajectory groups – ALL SAMPLE**

| **HRQOL** | % of population in each group  (n=2069) | BIC | AIC | Entropy |
| --- | --- | --- | --- | --- |
| 1 group, quartic | 100% | -56447.31 | -56430.40 | ---- |
| 2 groups, quartic | 30%; 70% | -54402.96 | -54369.15 | 0.861 |
| 3 groups, quartic | 9%; 40%; 51% | -53764.79 | -53714.08 | 0.840 |
| 4 groups, quartic | 4%; 20%; 42%; 33% | -53604.81 | -53537.20 | 0.777 |
| 5 groups, quartic | 5%; 7%; 18%; 42%; 27% | -53474.95 | -53390.43 | 0.777 |
| 6 groups, quartic | 2%; 20%; 7%; 8%; 40%; 21% | -53385.71 | -53284.29 | 0.773 |

***Sensitivity sample: Trajectory figures for each model**

**
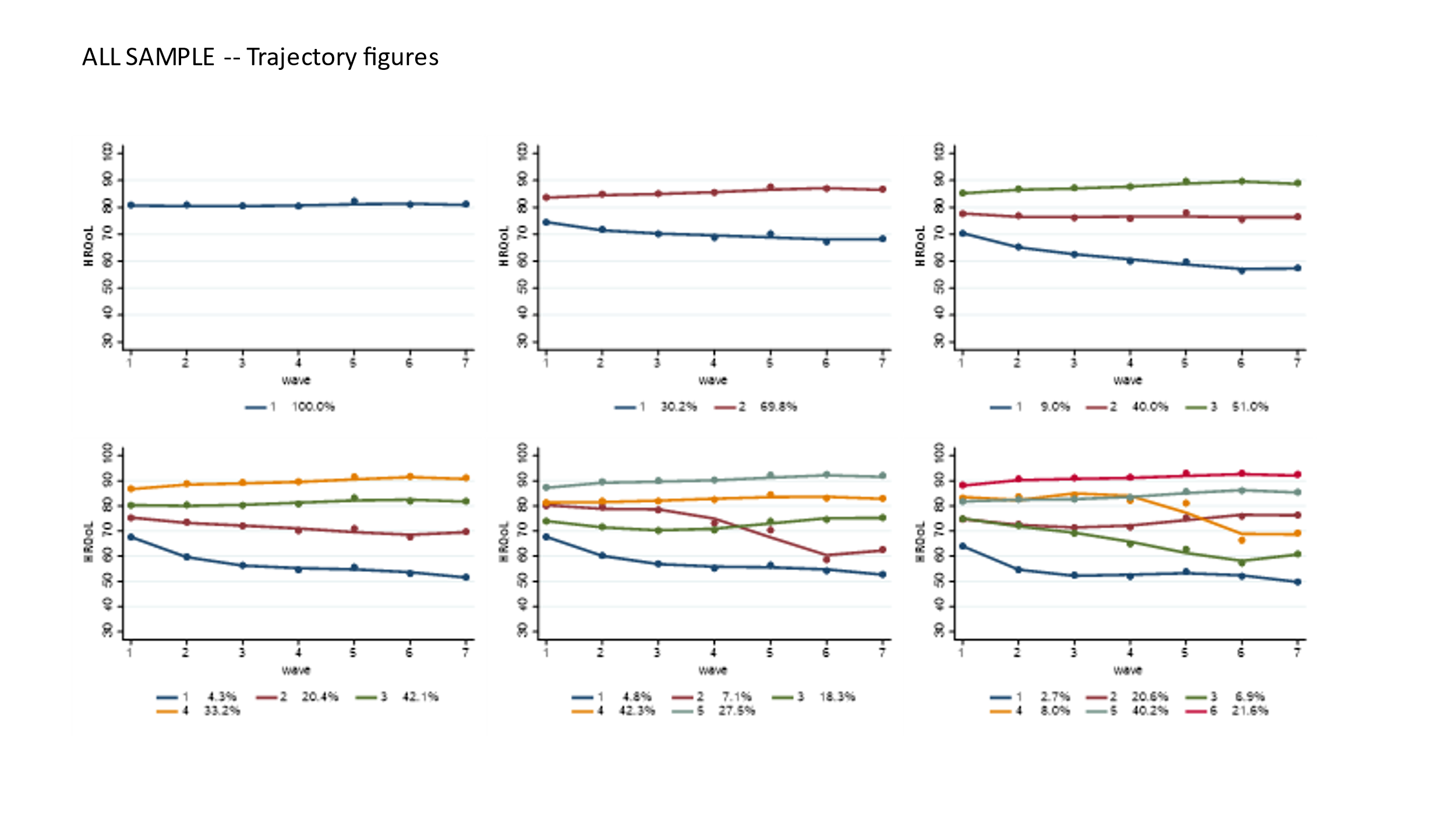
**

*** Sensitivity sample: Spaghetti plots for each model**

**
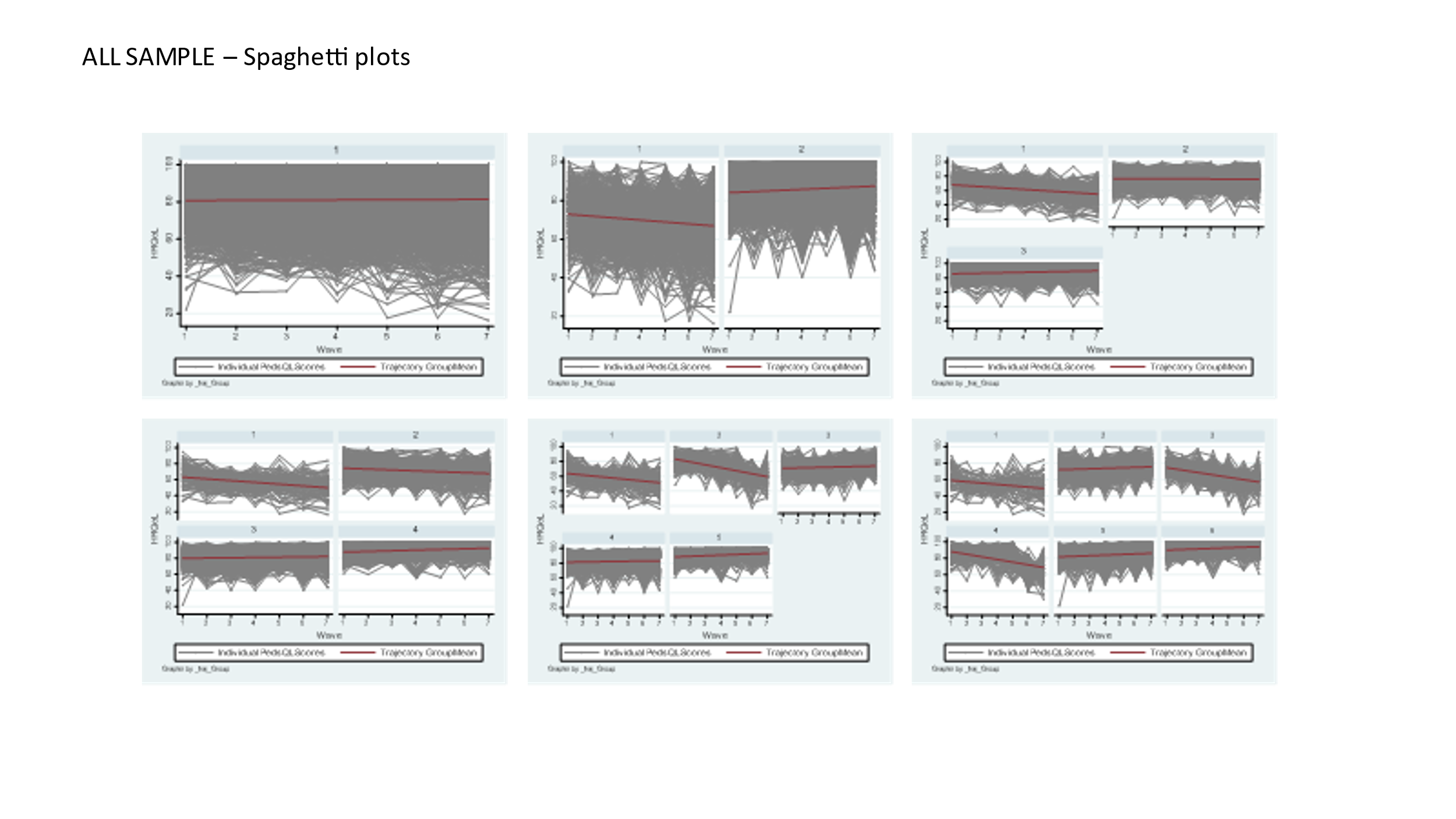
**

**Decision – 3-group model (* result = same as main analysis)**

- Sufficient entropy (>.80), BIC, AIC (rejected 4^th^ traj model because of this)
- Qualitatively distinct trajectories (rejected 4^th^ traj model because of this)
- Large enough groups (>5%; rejected 4^th^ and 6^th^ traj model because of this)
- Spaghetti plots cluster around mean

**Final 3-group model: Shape of trajectory groups – ALL SAMPLE**


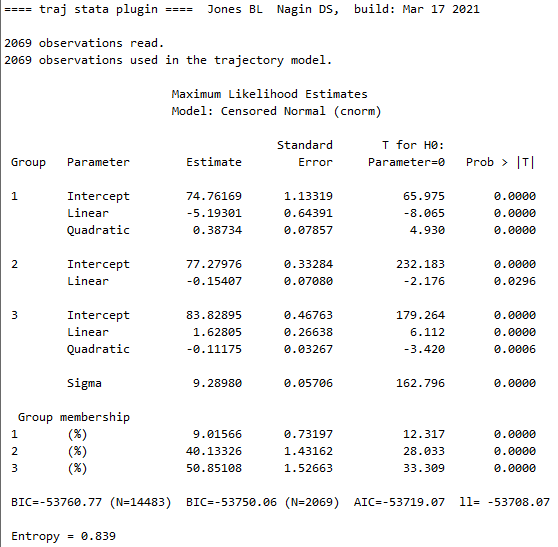

**Final 3-group model: posterior probabilities (PP) of individual membership within each group**

| **Trajectory Group – FOR ALL SAMPLE** | Obs. | Mean PP | SD | Min. | Max. |
| --- | --- | --- | --- | --- | --- |
| Group 1 | 183 | 0.94 | 0.10 | 0.52 | 1.00 |
| Group 2 | 822 | 0.91 | 0.13 | 0.50 | 0.99 |
| Group 3 | 1064 | 0.93 | 0.12 | 0.50 | 1.00 |

**Cross tab with original sample (using only those children included in both analyses)**


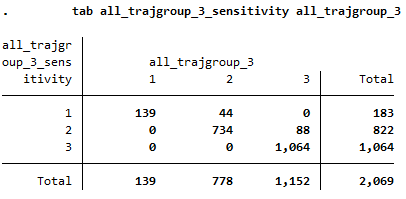


**4.2 SENSITIVITY ANALYSIS: BOYS**

**Decision statistics for the number and shape of trajectory groups – BOYS**

| **HRQOL** | % of population in each group  (n=1043) | BIC | AIC | Entropy |
| --- | --- | --- | --- | --- |
| 1 group, quartic | 100% | -28539.58 | -28524.73 | ---- |
| 2 groups, quartic | 25%; 75% | -27447.40 | -27417.71 | 0.884 |
| 3 groups, quartic | 10%; 41%; 48% | -27090.21 | -27045.66 | 0.839 |
| 4 groups, quartic | 4%; 15%; 42%; 38% | -27007.41 | -26948.02 | 0.818 |
| 5 groups, quartic | 4%; 7%; 13%; 41%; 34% | -26959.46 | -26885.21 | 0.803 |
| 6 groups, quartic | 3%; 6%; 6%; 29%; 38%; 18% | -26943.19 | -26854.09 | 0.767 |

***Sensitivity sample: Trajectory figures for each model**

**
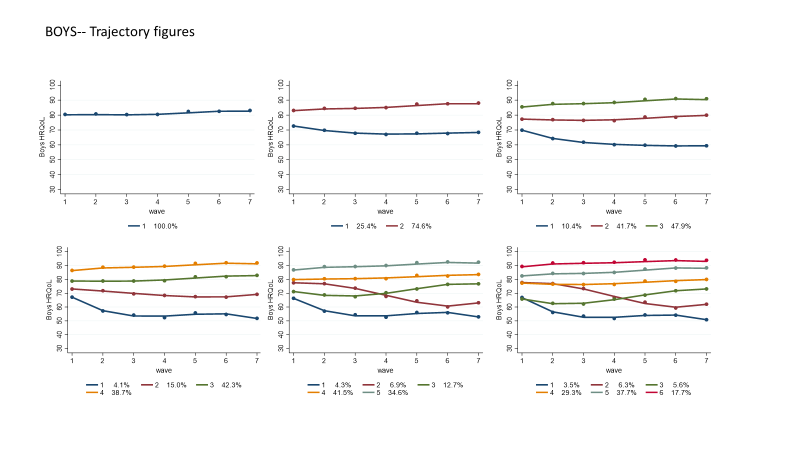
**

*** Sensitivity sample: Spaghetti plots for each model**

**
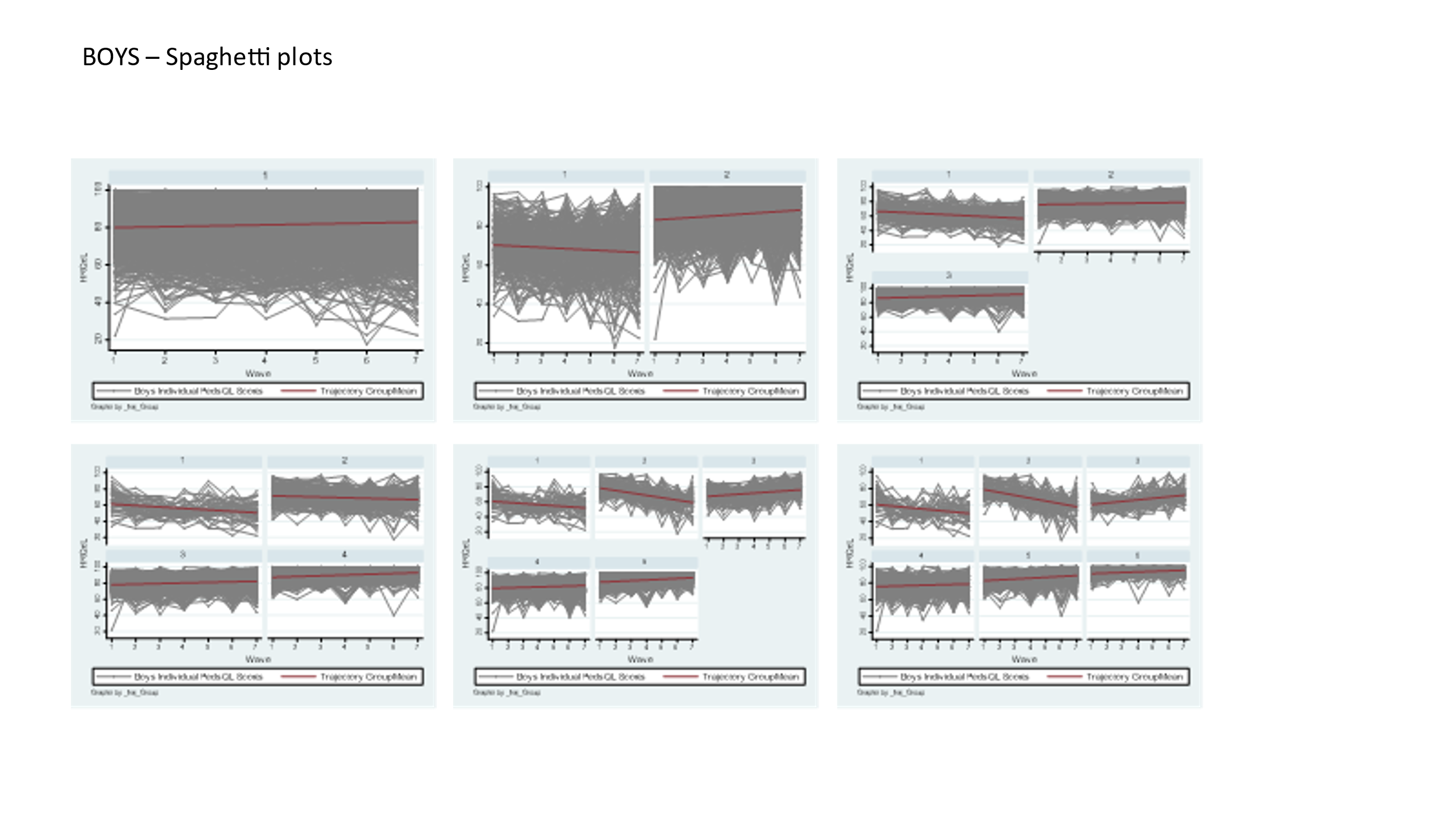
**

**Decision – 3-group model (* result = same as main analysis)**

- Sufficient entropy (>.80), BIC, AIC (rejected 6^th^ traj model because of this)
- Qualitatively distinct trajectories (rejected 4^th^ traj model because of this)
- Large enough groups (>5%; rejected 4^th^, 5^th,^ 6^th^ traj model because of this)
- Spaghetti plots cluster around mean


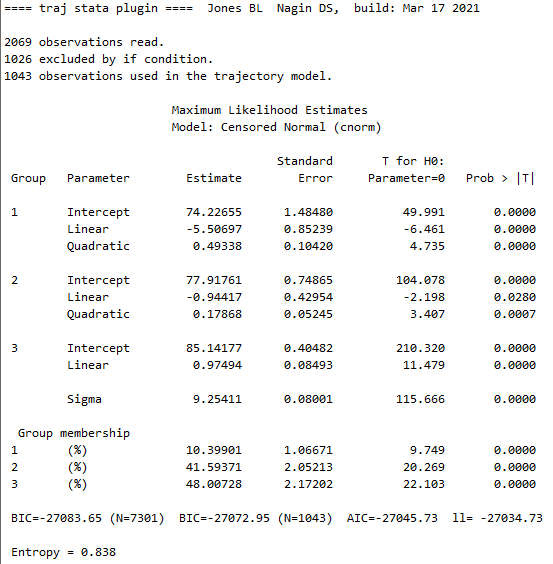
**Final 3-group model: Shape of trajectory groups – BOYS**

**Final 3-group model: posterior probabilities (PP) of individual membership within each group**

| **Trajectory Group – BOYS** | Obs. | Mean PP | SD | Min. | Max. |
| --- | --- | --- | --- | --- | --- |
| Group 1 | 104 | 0.97 | 0.08 | 0.56 | 1.00 |
| Group 2 | 436 | 0.91 | 0.13 | 0.51 | 0.99 |
| Group 3 | 503 | 0.93 | 0.12 | 0.50 | 1.00 |

**Cross tab with original sample (using only those children included in both analyses)**


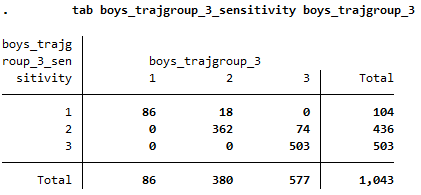


**4.3 SENSITIVITY ANALYSIS: GIRLS**

**Decision statistics for the number and shape of trajectory groups – GIRLS**

| **HRQOL** | % of population in each group  (n=1026) | BIC | AIC | Entropy |
| --- | --- | --- | --- | --- |
| 1 group, quartic | 100% | -27880.46 | -27865.66 | ---- |
| 2 groups, quartic | 35%; 65% | -26924.51 | -26894.91 | 0.849 |
| 3 groups, quartic | 7%; 39%; 53% | -26639.25 | -26594.85 | 0.858 |
| 4 groups, quartic | 5%; 30%; 44%; 20% | -26579.02 | -26519.82 | 0.769 |
| 5 groups, quartic | 2%; 8%; 31%; 44%; 15% | -26542.14 | -26468.14 | 0.784 |
| 6 groups, quartic | 2%; 7%; 25%; 42%; 8%; 16% | -26470.42 | -26381.62 | 0.787 |

***Sensitivity sample: Trajectory figures for each model**

**
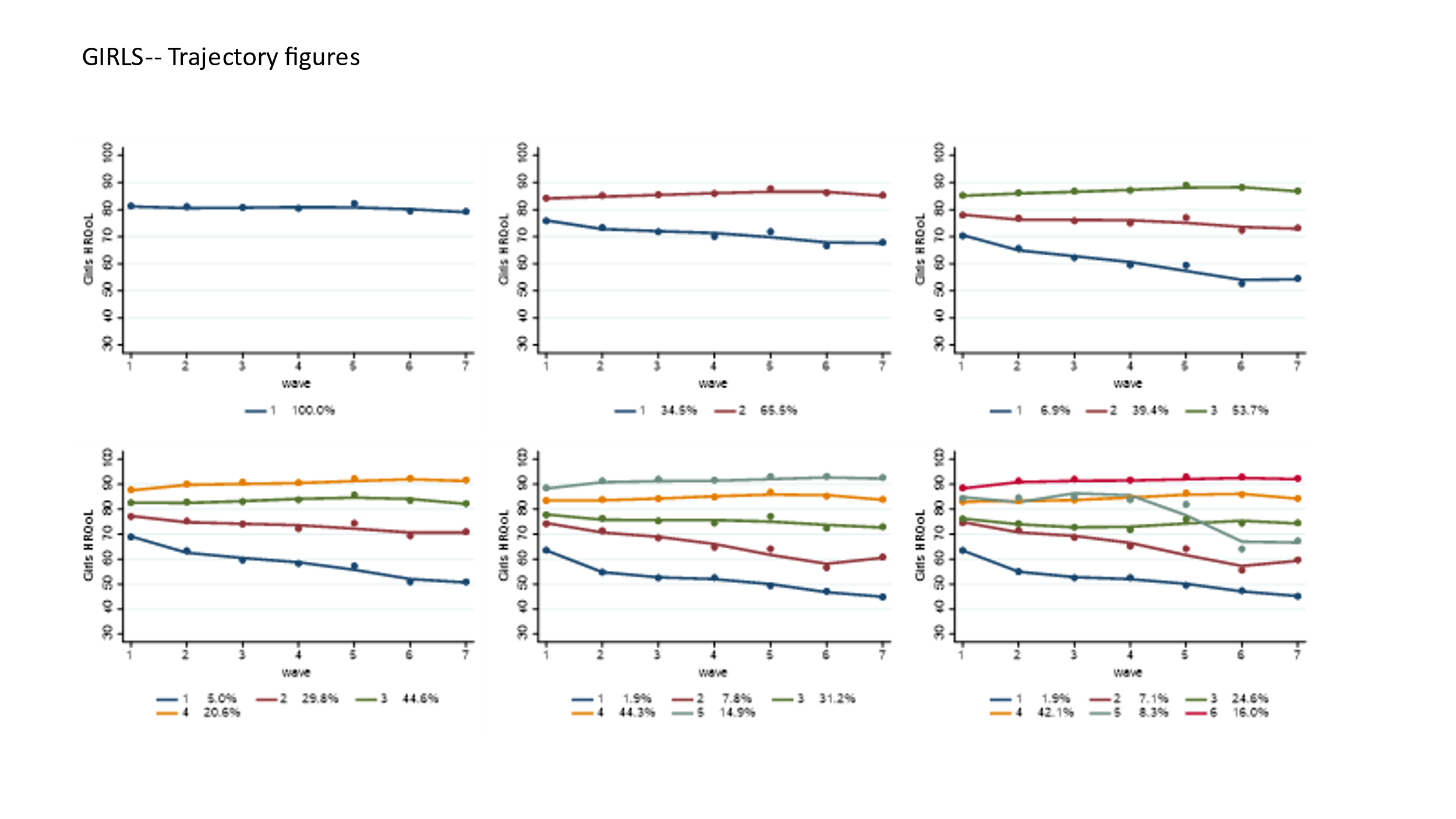
**

*** Sensitivity sample: Spaghetti plots for each model**

**
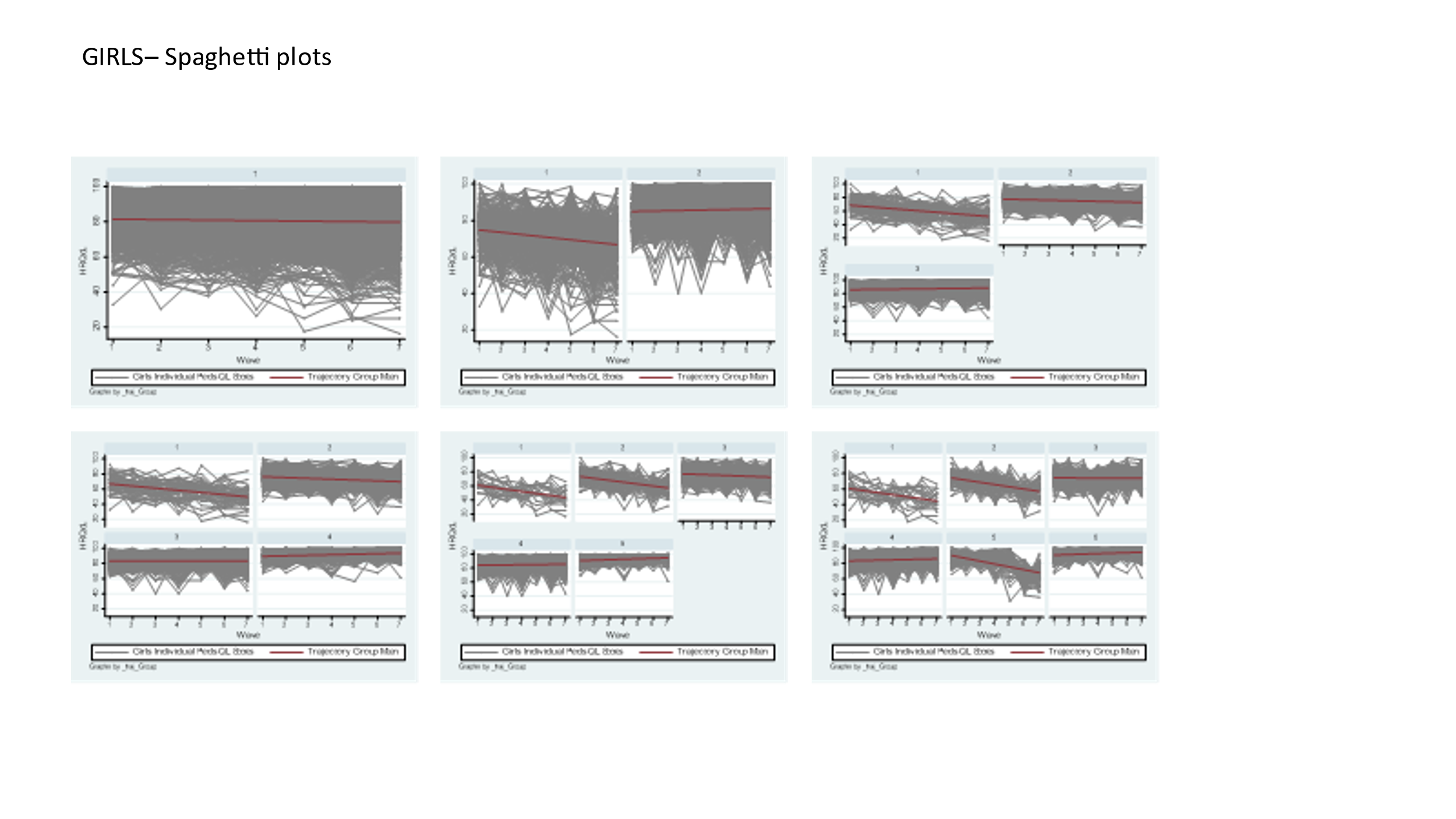
**

**Decision – 3-group model (* result = same as main analysis)**

- Sufficient entropy (>.80), BIC, AIC (rejected 4^th^ traj model because of this)
- Qualitatively distinct trajectories (rejected 4^th^ traj model because of this)
- Large enough groups (>5%; rejected 5^th,^ 6^th^ traj model because of this)
- Spaghetti plots cluster around mean


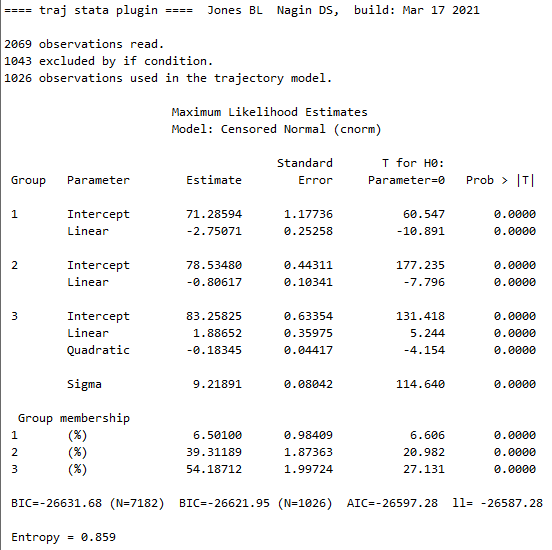
**Final 3-group model: Shape of trajectory groups – GIRLS**

**Final 3-group model: posterior probabilities (PP) of individual membership within each group**

| **Trajectory Group – GIRLS** | Obs. | Mean PP | SD | Min. | Max. |
| --- | --- | --- | --- | --- | --- |
| Group 1 | 64 | 0.92 | 0.14 | 0.52 | 1.00 |
| Group 2 | 404 | 0.92 | 0.13 | 0.50 | 0.99 |
| Group 3 | 558 | 0.95 | 0.10 | 0.50 | 1.00 |

**Cross tab with original sample (using only those children included in both analyses)**


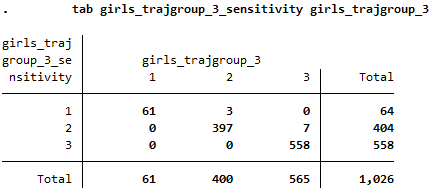


**REFERENCES FOR SUPPLEMENTARY MATERIAL**

1. Wallander JL, Fradkin C, Elliott MN, Cuccaro PM, Tortolero Emery S, Schuster MA. Racial/ethnic disparities in health-related quality of life and health status across pre-, early-, and mid-adolescence: a prospective cohort study. *Qual Life Res*. 2019;28(7):1761-1771. doi:10.1007/s11136-019-02157-1

2. Bronfenbrenner U. *The Ecology of Human Development: Experiments by Nature and Design.* Harvard University Press; 1979.

3. Varni JW, Burwinkle TM, Seid M, Skarr D. The PedsQL^TM^* 4.0 as a pediatric population health measure: Feasibility, reliability, and validity. *Ambul Pediatr*. 2003;3(6):329-341. doi:10.1367/1539-4409(2003)003<0329:TPAAPP>2.0.CO;2

4. Goodman R. The strengths and difficulties questionnaire: A research note. *J Child Psychol Psychiatry Allied Discip*. 1997;38(5):581-586. doi:10.1111/j.1469-7610.1997.tb01545.x

5. Hawes DJ, Dadds MR. Australian data and psychometric properties of the Strengths and Difficulties Questionnaire. *Aust N Z J Psychiatry*. 2004;38(8):644-651. doi:10.1111/j.1440-1614.2004.01427.x

6. Mellor D. Normative data for the Strengths and Difficulties Questionnaire in Australia. *Aust Psychol*. 2005;40(3):215-222. doi:10.1080/00050060500243475

7. Cole TJ, Bellizzi MC, Flegal KM, Dietz WH. Establishing a standard definition for child overweight and obesity worldwide: international survey. *BMJ*. 2000;320:1240. doi:10.1136/bmj.320.7244.1240

8. Kessler RC, Barker PR, Colpe LJ, et al. Screening for serious mental illness in the general population. *Arch Gen Psychiatry*. 2003;60(2):184-189. doi:10.1001/archpsyc.60.2.184

9. Bastiaansen D, Koot HM, Ferdinand RF. Determinants of quality of life in children with psychiatric disorders. *Qual Life Res*. 2005;14(6):1599-1612. doi:10.1007/s11136-004-7711-2

10. Farrant B. Maladaptive parenting and child emotional symptoms in the early school years: Findings from the Longitudinal Study of Australian Children. *Australas J Early Child*. 2014;39(2):118-125. doi:10.1177/183693911403900215

11. O’Loughlin R, Hiscock H, Pan T, Devlin N, Dalziel K. The relationship between physical and mental health multimorbidity and children’s health-related quality of life. *Qual Life Res*. 2022;(Epub ahead of print). doi:10.1007/s11136-022-03095-1

12. Jozefiak T, Wallander JL. Perceived family functioning , adolescent psychopathology and quality of life in the general population: a 6-month follow-up study. *Qual Life Res*. 2016;25(4):959-967. doi:10.1007/s11136-015-1138-9

13. Jozefiak T, Greger HK, Koot HM, Klöckner CA, Wallander JL. The role of family functioning and self-esteem in the quality of life of adolescents referred for psychiatric services: a 3-year follow-up. *Qual Life Res*. 2019;28(9):2443-2452. doi:https://doi.org/10.1007/s11136-019-02197-7

14. Le HND, Mensah F, Eadie P, et al. Health-related quality of life of children with low language from early childhood to adolescence: results from an Australian longitudinal population-based study. *J Child Psychol Psychiatry*. 2021;62(3):349-356. doi:10.1111/jcpp.13277

15. Mullan K, Daraganova G, Baker K. *Growing Up in Australia : The Longitudinal Study of Australian Children (LSAC). LSAC Technical Paper #14. Imputing Income in the Longitudinal Study of Australian Children.*; 2015.

16. Australian Bureau of Statistics. 2033.0.55.001 - Census of Population and Housing: Socio-Economic Indexes for Areas (SEIFA), Australia, 2016. Canberra: Australian Bureau of Statistics. Published 2018. Accessed March 23, 2021. https://www.abs.gov.au/ausstats/abs@.nsf/Lookup/by Subject/2033.0.55.001~2016~Main Features~IRSAD~20

17. Australian Bureau of Statistics. 1270.0.55.005 - Australian Statistical Geography Standard (ASGS): Volume 5 - Remoteness Structure, July 2016. Table 3: Correspondence 2017 Postcode to 2016 Remoteness Area. Canberra: Australian Bureau of Statistics. Published 2018. Accessed March 23, 2021. https://www.abs.gov.au/AUSSTATS/abs@.nsf/DetailsPage/1270.0.55.005July 2016?OpenDocument

18. Arefadib N, Moore T. *Reporting the Health and Development of Children in Rural and Remote Australia. Report for the Royal Far West.*; 2017.

19. Nagin DS, Odgers CL. Group-based trajectory modeling in clinical research. *Annu Rev Clin Psychol*. 2010;6:109-138. doi:10.1146/annurev.clinpsy.121208.131413

20. Hickson RP, Annis IE, Killeya-Jones LA, Fang G. Opening the black box of the group‐based trajectory modeling process to analyze medication adherence patterns: An example using real-world statin adherence data. *Pharmacoepidemiol Drug Saf*. 2020;29(3):357-362.

21. Vella SA, Magee CA, Cliff DP. Trajectories and Predictors of Health-Related Quality of Life during Childhood. *J Pediatr*. 2015;167(2):422-427. doi:10.1016/j.jpeds.2015.04.079
